# Improvement effect of biochar on soil microbial community structure and metabolites of decline disease bayberry

**DOI:** 10.3389/fmicb.2023.1154886

**Published:** 2023-05-12

**Authors:** Haiying Ren, Hao Guo, Mohammad Shafiqul Islam, Haitham E. M. Zaki, Zhenshuo Wang, Hongyan Wang, Xingjiang Qi, Junning Guo, Li Sun, Qi Wang, Bin Li, Gang Li, Khlode S. A. Radwan

**Affiliations:** ^1^State Key Laboratory for Managing Biotic and Chemical Treats to the Quality and Safety of Agro-Products, Institute of Horticulture, Institute of Agro-product Safety and Nutrition, Zhejiang Academy of Agricultural Sciences, Hangzhou, China; ^2^Xianghu Laboratory, Hangzhou, China; ^3^Department of Plant Pathology and MOA Key Lab of Pest Monitoring and Green Management, College of Plant Protection, China Agricultural University, Beijing, China; ^4^Institute of Biotechnology, Zhejiang University, Hangzhou, China; ^5^Horticulture Department, Faculty of Agriculture, Minia University, El-Minia, Egypt; ^6^Applied Biotechnology Department, University of Technology and Applied Sciences-Sur, Sur, Oman; ^7^Plant Pathology Department, Faculty of Agriculture, Minia University, El-Minia, Egypt

**Keywords:** bayberry, decline disease, biochar, vegetative growth, fruit quality, physical and chemical properties, microorganism, metabolite

## Abstract

Decline disease is a new disease that has recently caused severe damage in bayberry industry. The effect of biochar on decline disease was determined by investigating the changes in the vegetative growth and fruit quality of bayberry trees as well as soil physical and chemical properties, microbial community structure, and metabolites. Results indicated that the application of biochar could improve the vigor and fruit quality of diseased trees, and rhizosphere soil microbial diversity at the levels of phyla, orders, and genera. The relative abundance of *Mycobacterium*, *Crossiella*, *Geminibasidium*, and *Fusarium* were significantly increased, while *Acidothermus*, *Bryobacter*, *Acidibacter*, *Cladophialophora*, *Mycena*, and *Rickenella* were significantly decreased by biochar in rhizosphere soil of decline diseased bayberry. Analysis of redundancies (RDA) of microbial communities and soil characteristics revealed that the composition of bacterial and fungal communities was significantly affected by the pH, organic matter, alkali hydrolyzable nitrogen, available phosphorus, available potassium, exchangeable calcium and exchangeable magnesium in bayberry rhizosphere soil, and the contribution rates to fungi were larger than those to bacteria at the genus level. Biochar greatly influenced the metabolomics distribution of rhizosphere soils of decline disease bayberry. One hundred and nine different metabolites from both the presence and absence of biochar, mainly include acid, alcohol, ester, amine, amino acid, sterol, sugar, and other secondary metabolites, of which the contents of 52 metabolites were increased significantly such as aconitic acid, threonic acid, pimelic acid, epicatechin, and lyxose. The contents of 57 metabolites decreased significantly, such as conduritol β-expoxide, zymosterol, palatinitol, quinic acid, and isohexoic acid. There was a great difference between the absence and presence of biochar in 10 metabolic pathways, including thiamine metabolism, arginine and proline metabolism, glutathione metabolism, ATP-binding cassette (ABC) transporters, butanoate metabolism, cyanoamino acid metabolism, tyrosine metabolism, phenylalanine metabolism, phosphotransferase system (pts), and lysine degradation. There was a significant correlation between the relative content of microbial species and the content of secondary metabolites in rhizosphere soil at the levels of bacterial and fungal phyla, order, and genus. Overall, this study highlighted the significant influence of biochar in decline disease by regulating soil microbial community, physical and chemical properties, and secondary metabolites in rhizosphere soil, which provided a novel strategy for managing bayberry decline disease.

## 1. Introduction

*Myrica rubra* is an important fruit tree in the south of China, with a cultivation area of about 334,000 ha and an annual output of about 950,000 tons. It is also distributed in Japan, France, Kenya, the United States, and Brazil (Sun et al., 2013; Ren et al., 2019). It has high nutritional value and health care effects. Bayberry is rich in juice, sweet, and sour. It contains carbohydrates, organic acids, proteins, and other substances and is rich in vitamin C and anthocyanins. At the same time, it is also an important medicinal plant. Its extract contains antioxidants, which can fight inflammation, allergy, diabetes, cancer, bacterial infection, diarrhea, and so on (Sun et al., 2013; Ren et al., 2019). However, in recent years, a major disease namely decline disease has occurred in the bayberry production area, mainly in orchards during the peak production period, which is manifested in the deterioration of tree growth, decline in fruit quality, relatively poor trace elements in the soil, and possible excess of available phosphorus (Ren et al., 2021a). The development of effective technology to rejuvenate diseased trees has become an urgent industry demand, which can also provide a theoretical basis for the healthy development of the bayberry industry.

The increase in crop production demand leads to excessive application of chemical fertilizers and pesticides in agriculture, which cause soil hardening and acidification, poor plant growth, and severe environmental pollution (Blanco-Canqui and Schlegel, 2013). Biochar is a carbon-rich solid substance produced by pyrolysis of crop straw, wood, livestock, and poultry manure under anoxic conditions, which plays a crucial role in environmental protection, forestry, and agriculture (Antal and Grønli, 2003). Furthermore, biochar can adsorb heavy metals in soil, reduce its toxicity to soil microbial community, and thus improve soil microbial and biochemical functions under heavy metal stress (Jing et al., 2018; Andrey et al., 2019; Varjani et al., 2019). In addition, biochar can improve fertility by changing acidity, porosity, cultivation intensity, and physical and chemical properties of soil (De Tender et al., 2016; Novak et al., 2016; Frenkel et al., 2017; Jaiswal et al., 2018; Ibrahim et al., 2019). Biochar also plays a vital role in the prevention and control of diseases, such as soybean root rot (Rogovska et al., 2017), cucumber rot (Jaiswal et al., 2019), cucumber collapse (Jaiswal et al., 2014), bayberry decline disease (You et al., 2021), powdery mildew of leaves (Elad et al., 2011), and soil-borne root rot diseases (Bonanomi et al., 2015).

On the other hand, biochar also can adjust the structure of microbial community (Hu et al., 2014) and improve soil microbial diversity (Elmer and Pignatello, 2011), promote the growth of beneficial microorganisms such as *Pseudomonas* and *Trichoderma* (Graber et al., 2010). Soil microorganism is a vital ecosystem component, and their activity can reflect soil fertility (Zhou et al., 2014). Furthermore, the metabolites produced by various soil microorganism have been reported to be involved in plant growth, development, defense, and other physiological processes. Therefore, removing toxic root metabolites will help to improve the poor growth environment of plants, while making full use of soil metabolites beneficial to plants will help to cultivate healthy plants, and the change of soil metabolites will also have a particular impact on the structure of the soil microbial community.

In order to find an alternate method to prevent and control bayberry decline disease, this study investigated the effect of biochar on tree growth, fruit quality, soil physical and chemical properties, soil microorganisms, and soil metabolites. The result of this study will provide a scientific basis for developing improved soil technology around the root of diseased bayberry’s decline and promoting the bayberry industry’s sustainable development.

## 2. Materials and methods

### 2.1. Experimental design

In July 2018, 15-year-old Dongkui bayberry, whose leaf abscission amount accounted for about 50% of the total leaf amount of the tree in Dongfanghe Village, Majian Town, Lanxi City, was used as the test material, with disease index ranging from grade 1 to 9. Row spacing of 4 m × 5 m, the bayberry trees with similar load, crown size, and leaf abscission accounted for 10–25% of the whole leaves (disease index was level 3) are selected as the tested trees, and the tested park was conventional managed (Ren et al., 2022b). After fruit picking, 20 kg/plant biochar fertilizer (25–32% pig dung, 18–24% biochar, 15–21% potassium sulfate, 8–12% urea, 3–6% mono-ammonium phosphate, 8–10% molasses, 1–1.5% chitosan, 0.5–1% borax, and 6–8% weathered coal) were applied to the root drip line ditch once. The decrease of diseased bayberry trees without fertilization was used as the control (CK). Each tree was counted as one repetition, and every 15 trees were processed. The spring shoots, leaves, fruits, and rhizosphere soil of bayberry were collected in June 2019 to determine various indicators. The 2 kg of 0–20 cm topsoil samples were collected with the quartering method about 1 m around the bayberry trunk at the crown drip line. Some were stored in a − 20°C refrigerator for DNA extraction. Some were dried naturally at room temperature and passed through a 40-mesh screen to detect soil nutrients.

### 2.2. Measurement of vegetative growth parameters

The collection method and parameter detection method of branch and leaf samples referred to the previous paper published by Ren et al. (2021a,b). Six diseased bayberry plants were treated either with or without biochar. Five spring shoots were randomly tested for each tree in the east, south, west, and north directions, and 30 shoots were taken for with or without biochar, respectively. The length and diameter of shoots were measured with a digital vernier caliper (Shanghai Daoju, China). Thirty leaves were selected randomly for with or without biochar, with five leaves for each tree, which were taken from below the top of the vegetative branches at the middle periphery of the tree body. The chlorophyll mass fraction (SPAD value) was measured by using the SPAD-502 PLus chlorophyll meter (Japanese Minolta Company), while leaf length (top to petiole base) and leaf width were measured with a digital vernier caliper. The thickness of 10 leaves was measured every time with a digital vernier caliper, and the average of the 30 leaves was determined by measuring three times.

### 2.3. Determination of fruit characters of bayberry

The collection and detection method of fruit samples referred to the previous paper published by Ren et al. (2021a,b). Two hundred fruits from each direction were sampled and transported to the laboratory the day after collection. The single fruit weight, soluble solids, and hardness were measured immediately, and the others were stored at −20°C for the determination of total sugar and titratable acid content. Each parameter was from 15 fruits, respectively. The single fruit weights were tested with an electronic balance (Shanghai Precision Instrument), and the content of soluble solids (TSS) with ATAGOPR-101a handheld digital sugar glucometer (Japan). The fruit hardness was measured with TA-XT plus texture analyzer (TA-MTP was selected as the probe, and the pressing distance was 4.0 mm, unit: g/cm^2^). Titrable acid was measured by acid–base titration. The average values of each parameter were taken.

### 2.4. Determination of soil physical and chemical properties

The collection method of soil samples and the detection method of physical and chemical indexes refer to the previous paper published by Ren et al. (2021a,b). The rhizosphere soil samples from each of the six trees in each treatment were taken 1 week after the fruits were picked from trees with stable vegetative and reproductive growth. About 2 kg of mixed soil samples (0–20 cm) were taken from the drip line around the crown of the bayberry plant with the quartering method and put through a 0.45-mm sieve.

One-half sample was kept in a refrigerator at −80°C for the DNA extraction, and the other was air dried at room temperature to measure the soil’s physical and chemical properties. The organic matter content was tested using the K_2_Cr_2_O_7_ oxidation method, and the pH was measured using a pH meter with a soil-to-water ratio of 1:2.5 (Pan et al., 2018). Available N was tested with the alkaline hydrolysis diffusion method; available P was evaluated with anti-molybdenum antimony colorimetry (Shibl et al., 2020); available K was determined by ammonium acetate extraction flame photometer method and exchangeable calcium and magnesium were tested with an ice3500 atomic absorption spectrophotometer after being extracted with ammonium acetate and iron.

### 2.5. Determination of soil flora

The soil sample processing and microbiome sequencing refer to Ren et al. (2021a,b). The genome sequencing was entrusted to Shanghai Ouyi Biomedical Technology Co., Ltd. The total DNA of soil microorganisms was extracted; refer to the operation process of the kit manual. Using the extracted genomic DNA as the template, according to the sequencing region, select specific primers with Barcode and Takara ExTaq high fidelity enzyme of Takara Company for PCR amplification. The corresponding region of bacterial diversity identification: 16S rRNA V3–V4 region (primer 343F and 798R), front-end primer 343F-5′-TACGGRAGGCAG-3′, and back-end primer: 798R-5′-AGGGTATCTAATCCT-3′ PCR (Nossa et al., 2010). The corresponding fungal ITS diversity identification regions were ITS1 and ITS2 (primer ITS1F and ITS2), primer sequence ITS1F-5′-CTTGGTCATTTAGAGGAAGTAA-3′, backend primer: ITS2-5′-GCTGCGTTCATCGATGC-3′ (Mukherjee et al., 2014). The components of the PCR included 1 μL DNA template, 1 μL (10 μM) each forward and reverse primer, 15 μL 2 × Hieff® Robust PCR Master Mix, and 12 μL ddH2O. The PCR amplicons were purified with Vazyme V AHTSTM DNA clean beads (Vazyme, Nanjing, China). Afterward, amplicons with equal amounts were pooled, and 2 × 250 bp pair-end sequencing was accomplished through the Illumina MiSeq system [Sangon Biotech (Shanghai) Co., Ltd., Shanghai, China].

### 2.6. Determination of root exudates

The methods of soil sample processing and secondary metabolite detection refer to the author’s previously published paper (Ren et al., 2022b). In detail, one quality control (QC) sample was added to every 10 samples to demonstrate that the whole analysis was accurate. The QC sample was made by mixing the extracts of all the samples in the same amount, and each QC sample was the same size as the tested sample. Also, the soil samples were kept at room temperature for GC–MS metabolomics analysis. This test was done on a 7890B-5977A GC/MSD GC–MS (Agilent Technologies Inc., Santa Clara, CA, United States) as our recent publication (Ren et al., 2022b). In this study, the data were analyzed by comparing them to the National Institute of Standards and Technology’s standard spectrum library. The information about metabolites was looked up in the KEGG database.

### 2.7. Data analysis

T-test was used to compare the significant difference in alpha diversity using specific packages in R version 4.2.0 (The R Foundation for Statistical Computing, Vienna, Austria; Team 2013). Permutational Multivariate ANOVA (PERMANOVA) was used to examine whether the sample groups harbored significantly different microbial communities (i.e., beta diversity) in R. Redundancy discriminant analysis (RDA) was performed by using Canoco version 5.02. The collected data were compared to the National Institute of Standards and Technology’s standard spectrum library (NIST). Information about metabolites was looked up in the Kyoto Encyclopedia of Genes and Genomes (KEGG database). In addition, the heat map was drawn by using the R package, Pheatmap version 1.0.12 (https://CRAN.R-project.org/package=pheatmap, accessed on 15 October 2022). Orthogonal partial least squares discriminant analysis (OPLS-DA) was made by using R 3.6.2 ropls. With the support of Excel and Photoshop, a three-line version of the paper’s table was created.

## 3. Results

### 3.1. Effect of biochar on vegetative growth and fruit quality of decline diseased trees

Compared with the decline of diseased trees, all the vegetative growth parameters after the application of biochar were improved, in which the branch length, branch diameter, leaf length, leaf width, leaf thickness, and chlorophyll content were significantly increased by 56.65, 57.56, 17.46, 28.45, 17.62, and 26.65%, respectively. Furthermore, significant changes were also observed in the quality parameters of the bayberry fruit of the declining diseased tree. Compared with the disease control, the weight, titratable acid, and total sugar content of fruit were significantly increased by 22.05, 50.00, and 32.26%, respectively (Table 1).

**Table 1:** Effects of biochar on vegetative growth and fruit quality of decline diseased bayberry.

Parameter (mm)	D	B	Parameters	D	B
**Vegetative growth**					
Branch length	56.82 ± 1.50	89.01 ± 6.85^*^	Leaf width (mm)	27.21 ± 1.00	34.95 ± 1.86^*^
Branch diameter	2.05 ± 0.12	3.23 ± 0.12^*^	Leaf thickness (10 pieces/mm)	4.20 ± 0.10	4.94 ± 0.34^*^
Leaf length	90.15 ± 1.25	105.89 ± 3.01^*^	Chlorophyll content (SPAD)	35.05 ± 2.57	44.39 ± 0.83^*^
**Fruit quality**					
Titrable acid (%)	0.24 ± 0.02	0.36 ± 0.01^*^	Single fruit weight (g)	26.49 ± 2.93	32.33 ± 2.35^*^
Total sugar (%)	10.60 ± 1.32	14.02 ± 0.73^*^			

D and B represent the decline of diseased bayberry in the absence and presence of biochar, respectively. “^*^” shows significant increases compared to the control of the decline of diseased trees (*p* < 0.05).

### 3.2. Analysis of root-soil microbial community structure

**Figure 1:**
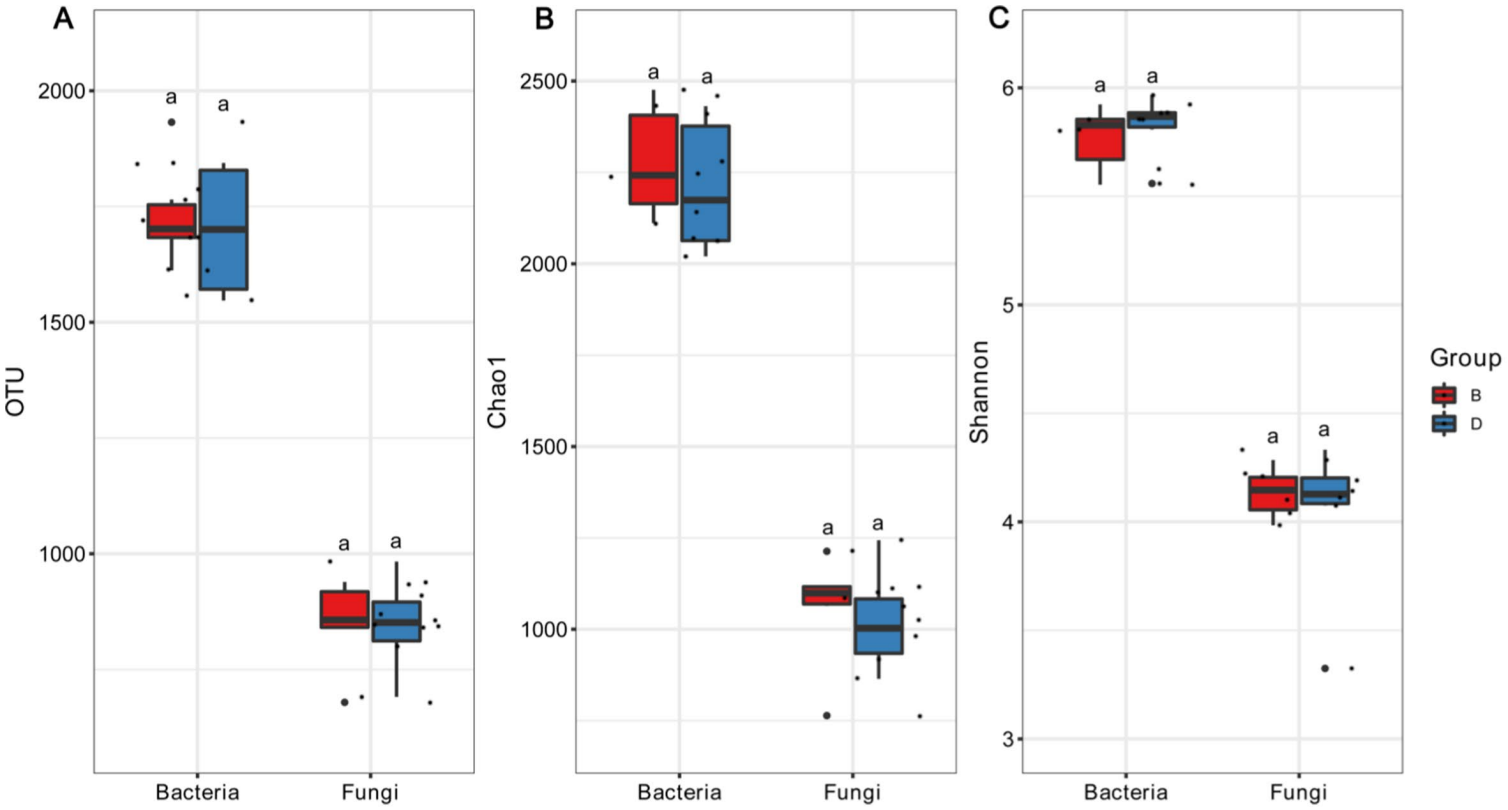
The influence of biochar on the OTU **(A)**, Chao1 **(B)**, and Shannon **(C)**. D and B represent declining diseases in absence and presence of biochar. Values with distinct lowercase letters within the same treatment indicate significant changes (*p* < 0.05).

There was a difference in the number of operational taxonomical units (OTUs) between the presence and the absence of biochar in the bacterial V3 + V4 region and fungal ITS region of the decline disease bayberry trees. In fact, the average number of bacterial OTUs in the absence of and presence of biochar were 1352.33 (range: 1,202–1,497) and 1385.50 (range: 1,264–1,585), respectively (Figure 1), while the number of bacterial OTUs in the rhizosphere soil of decline diseased trees in the presence of biochar was 2.45% higher than that in the absence of biochar. The average number of fungal OTUs in decline diseased bayberry trees were 750.67 (varying from 594 to 886) and 754.00 (varying from 582 to 842), respectively, in the absence and presence of biochar (Figure 1). In contrast, the number of fungi OTUs of rhizosphere soil in declining diseased bayberry trees was 0.44% higher in the presence of biochar than that in the absence of biochar. Therefore, it suggests that biochar had a greater effect on the bacterial operational taxonomic units than that of fungi of rhizosphere soil in declining diseased bayberry trees. Compared with the decline in diseased trees, the application of biochar caused a 3.51 and 0.96% increase, respectively, in the Chao1 index and Shannon index of bacteria; a 1.70 and 2.50% increase, respectively, in the Chao1 index and Shannon index of fungi in the rhizosphere of bayberry (Figure 1; Table 2).

**Table 2:** Physical and chemical properties of soil treated with biochar.

Parameters	D	B
pH value	4.41 ± 0.17	4.62 ± 0.10
Organic matter (%)	2.91 ± 0.20	3.32 ± 0.12^*^
Alkali hydrolyzed nitrogen (mg/kg)	84.63 ± 5.10	117.70 ± 9.16^*^
Available phosphorus (mg/kg)	205.71 ± 11.73	86.87 ± 10.57^#^
Available potassium (mg/kg)	432.22 ± 33.81	287.07 ± 4.77^#^
Exchangeable calcium (mg/kg)	348.63 ± 63.41	532.87 ± 117.10^*^
Exchangeable magnesium (mg/kg)	62.03 ± 1.39	98.27 ± 6.67^*^

D and B represent the decline of diseased bayberry in the absence and presence of biochar, respectively. ^*^ and ^#^ show significantly increased or reduced compared to the control of the decline diseased trees (*p* < 0.05).

### 3.3. Effect of biochar in soil microbial community structure

**Figure 2:**
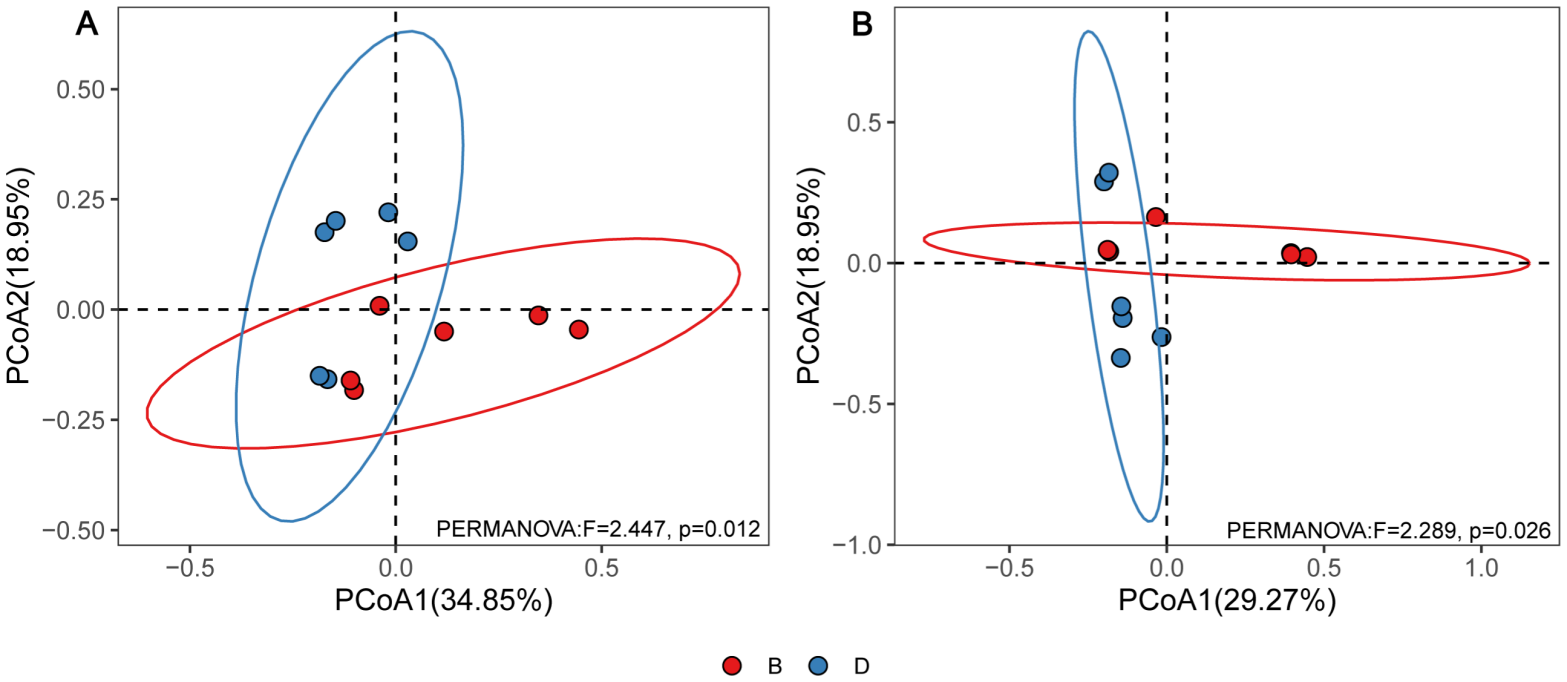
PcoA results **(A)** for soil bacteria and **(B)** for soil fungi based on OTU abundance. D and B represent the absence and presence of biochar in declining diseased bayberry trees, respectively.

According to the principal coordinate’s analysis (PCoA) of the bacterial community structure, the six replicates of the disease control and biochar treatments were split into two groups. In contrast, disease control was well separated from biochar treatments, demonstrating that biochar considerably affected the bacterial community structure of rhizosphere soil (Figure 2A). Furthermore, PCoA analysis of fungal community structure suggested that six replicates of the disease control and biochar treatment were divided into two distinct groups (Figure 2B). In addition, six replicates of the biochar treatments revealed a better diversity of bacterial community structure than those of fungal community structure (Figure 2).

**Figure 3:**
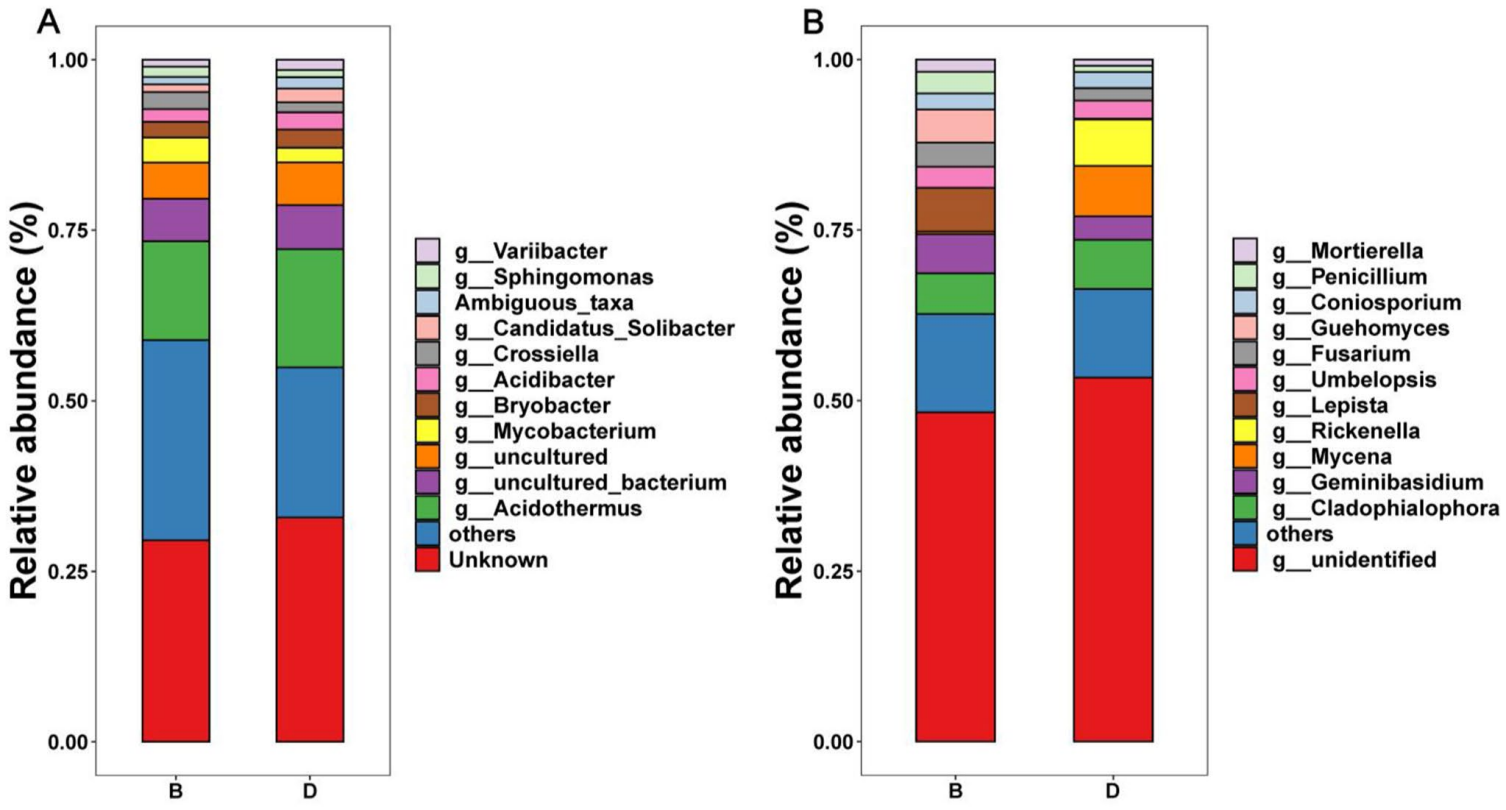
Relative abundance of bacteria **(A)** and fungi **(B)** at the genus level. D and B represent the absence and presence of biochar in diseased bayberry trees, respectively.

There was a significant difference in the relative abundance of the dominant bacteria and fungi at the phylum (Supplementary Figure S1), order (Supplementary Figure S2), and genus (Figure 3) level in the decline diseased bayberry rhizosphere soil between the absence and presence of biochar. Indeed, *Acidothermus*, *Mycobacterium*, *Bryobacter*, *Acidibacter*, and *Crossiella* were the main dominant bacterial groups at the level of genus in the top 5 of the relative abundance of bayberry rhizosphere soil, accounting for more than 95% of the total bacterial sequences (Figure 3A). *Cladophialophora*, *Geminibasidium*, *Mycena*, *Fusarium*, *Coniosporium*, and *Rickenella* were the prominent fungal genera (average relative abundance > 1%; Figure 3B).

### 3.4. Effects of biochar on rhizosphere soil physical and chemical properties

The application of biochar had a differential effect on the physical and chemical properties of rhizosphere soil of the declined bayberry. Compared with the diseased trees, the contents of organic matter, alkali hydrolyzed N, exchangeable Ca, and exchangeable Mg in the rhizosphere soil of the declined bayberry after biochar treatment were significantly increased by 14.09, 39.08, 52.85, and 58.42%, respectively. In contrast, the application of biochar caused a significant reduction in the contents of available P (57.77%) and available K (33.58%), respectively. In addition, no significant change was found on the pH (Table 3).

**Table 3:** Contribution of soil environment to bacteria and fungi taxa at the genus level.

Soil environment	Contribution at the bacterial genus level (%)	Contribution at the fungal genus level (%)
pH	7.0	11.4
Organic matter	7.3	12.5
Alkali hydrolyzed nitrogen	18.0	18.3
Available phosphorus	12.8	17.1
Available potassium	11.7	17.3
Exchangeable calcium	13.3	15.4
Exchangeable magnesium	12.1	17.4

### 3.5. Effect of biochar on RDA of soil properties and microbial communities

**Figure 4:**
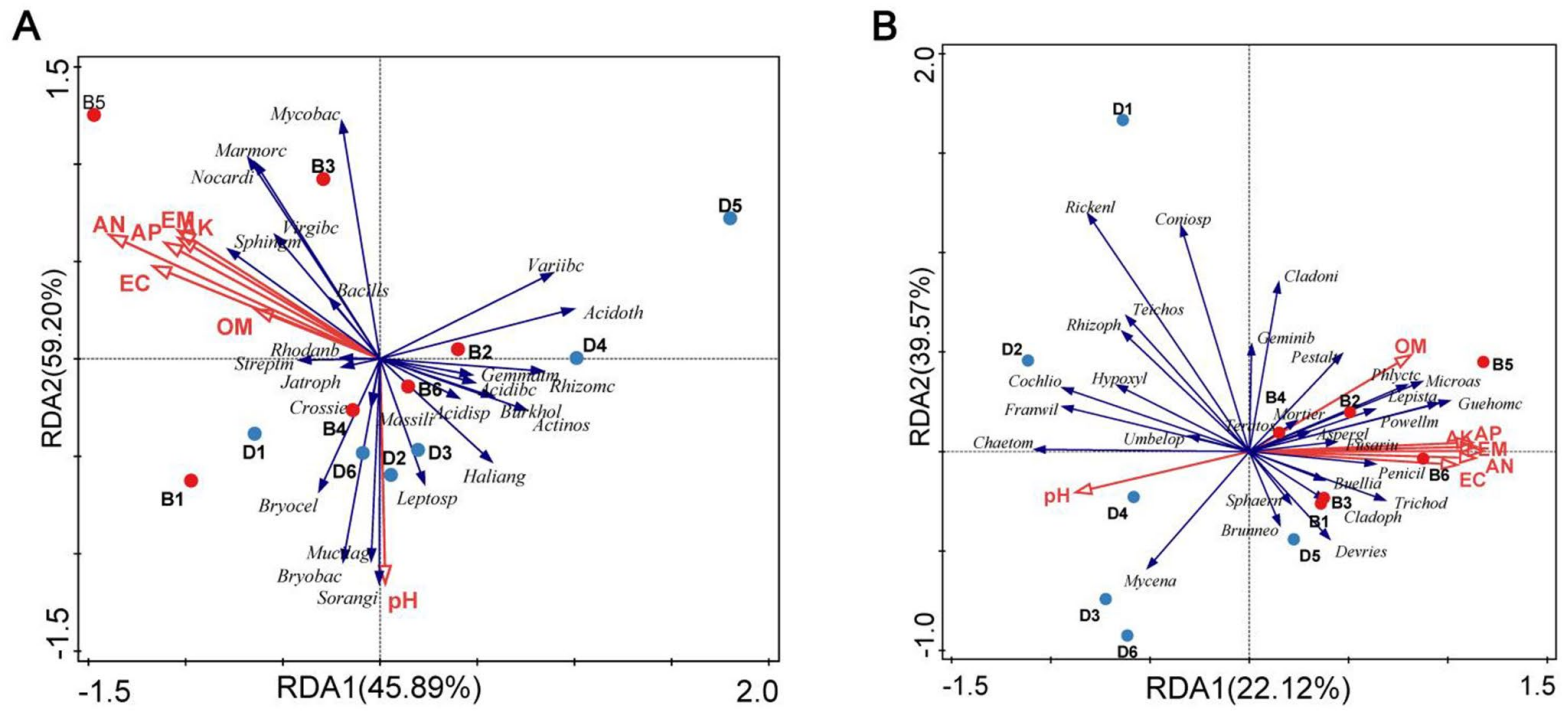
RDA of the rhizosphere microbial community composition at genus levels with soil physiological and chemical properties. RDA, redundancy discriminant analysis; PH, pH; OM, organic matter; AN, alkali-hydrolyzable nitrogen; AP, available phosphorus; AK, available potassium; EC, exchangeable calcium; EM, exchangeable magnesium; Blue dot: samples from Decline bayberry; Red Dot: sample from the biochar treated trees; D1–6 and B1–6 represented the absence and presence of the biochar in diseased bayberry trees. **(A)** Baceria. *Acidibc*: *Acidibacter*; *Acidisp*: *Acidisphaera*; *Acidoth*: *Acidothermus*; *Actinos*: *Actinospica*; *Bacills*: *Bacillus*; *Bryobac*: *Bryobacter*; *Bryocel*: *Bryocella*; *Burkhold*: *Burkholderia*; *Crossie*: *Crossiella*; *Gemmatm*: *Gemmatimonas*; *Haliang*: *Haliangium*; *Jatroph*: *Jatrophihabitans*; *Leptosp*: *Leptospirillum*; *Marmorc*: *Marmoricola*; *Massili*: *Massilia*; *Mucilag*: *Mucilaginibacter*; *Mycobac*: *Mycobacterium*; *Nocardi*: *Nocardioides*; *Rhizomc*: *Rhizomicrobium*; *Rhodanb*: *Rhodanobacter*; *Sphingm*: *Sphingomonas*; *Streptm*: *Streptomyces*; *Variibc*: *Variibacter*; *Virgibc*: *Virgibacillus*. **(B)** Fungi. *Aspergl*: *Aspergillus*; *Brunneo*, *Brunneosphaerella*; *Buellia*, *Buellia*; *Chaetom*: *Chaetomium*; *Cladoni*: *Cladonia*; *Cladoph*: *Cladophialophora*; *Cochlio*: *Cochliobolus*; *Coniosp*: *Coniosporium*; *Devries*: *Devriesia*; *Franwil*: *Franwilsia*; *Fusariu*: *Fusarium*; *Geminib*: *Geminibasidium*; *Guehomc*: *Guehomyces*; *Hypoxyl*: *Hypoxylon*; *Lepista*: *Lepista*; *Microas*: *Microascus*; *Mortier*: *Mortierella*; *Mycena*: *Mycena*; *Penicil*: *Penicillium*; *Pestalt*: *Pestalotiopsis*; *Phlyctc*: *Phlyctochytrium*; *Powellm*: *Powellomyces*; *Rhizoph*: *Rhizophlyctis*; *Rickenl*: *Rickenella*; *Sphaern*: *Sphaeronaemella*; *Teichos*: *Teichospora*; *Teratos*: *Teratosphaericola*; *Trichod*: *Trichoderma*; *Umbelop*: *Umbelopsis*.

In the RDA result, the first and second axes explained 45.89 and 59.20% of the cumulative variance of the rhizosphere microbial community-factor correction, respectively, at the bacterial genus level (Figure 4A), 22.12 and 39.57% of the cumulative variance of the rhizosphere microbial community-factor correction, respectively, at the fungal genus level (Figure 4B). The pH, organic matter, alkali hydrolyzable nitrogen, available phosphorus, available potassium, exchangeable calcium, and exchangeable magnesium in the rhizosphere soil of the decline bayberry treated with biochar had the large contribution rates to bacteria and fungi, the contribution rate to bacteria and fungi ranged from 7.0 to 18.0% and from 11.4 to 18.3%, respectively, and the contribution rate of alkali hydrolyzable nitrogen to bacteria and fungi were the largest, and all the contribution rates to fungi larger than those to bacteria (Table 3).

### 3.6. Change in rhizosphere soil metabolomics

**Figure 5:**
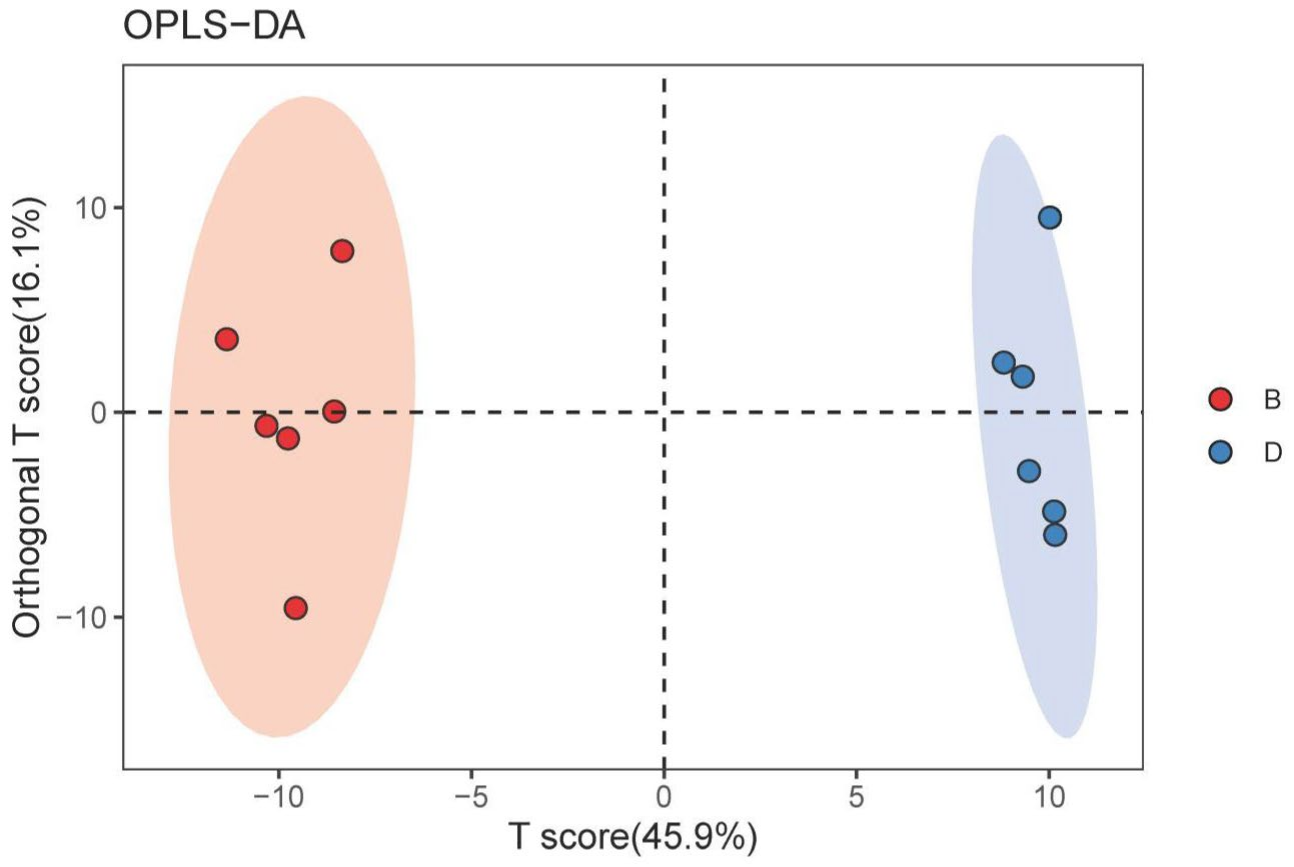
OPLS-DA score map of bayberry rhizosphere soil of the biochar treatment. D and B represent the biochar’s absence and presence in diseased bayberry trees, respectively.

A score map of metabolites was made using orthogonal partial least squares-discriminant analysis (OPLS-DA, a supervised statistical method of discriminant analysis), which can reduce intragroup differences, increase intergroup differences, and get rid of the impact of irrelevant factors on the experimental data to make accurate predictions of different samples. According to the findings, biochar greatly influenced the metabolomics distribution of rhizosphere soils from the decline disease bayberry. Indeed, in the absence of biochar, the declined disease samples were distributed in the positive area of *t* [1] (principal component 1). In contrast, in the presence of biochar, the declined disease samples were distributed in the negative area of *t* [1] (Figure 5). Furthermore, the parameters of the OPLS-DA are *R*^2^X(*cum*) = 0.62, *R*^2^Y(*cum*) = 0.993, Q^2^(*cum*) = 0. 972. The *Q*^2^ values (>0.5) showed that the model had good interpretation and prediction ability, which were good due to that the *Q*^2^ values were greater than 0.5. The cluster separation effect of D and B indicated that the application of biochar significantly changed the metabolic structure of the rhizosphere soil of the decline-diseased bayberry.

### 3.7. Analysis of differential metabolites

**Figure 6:**
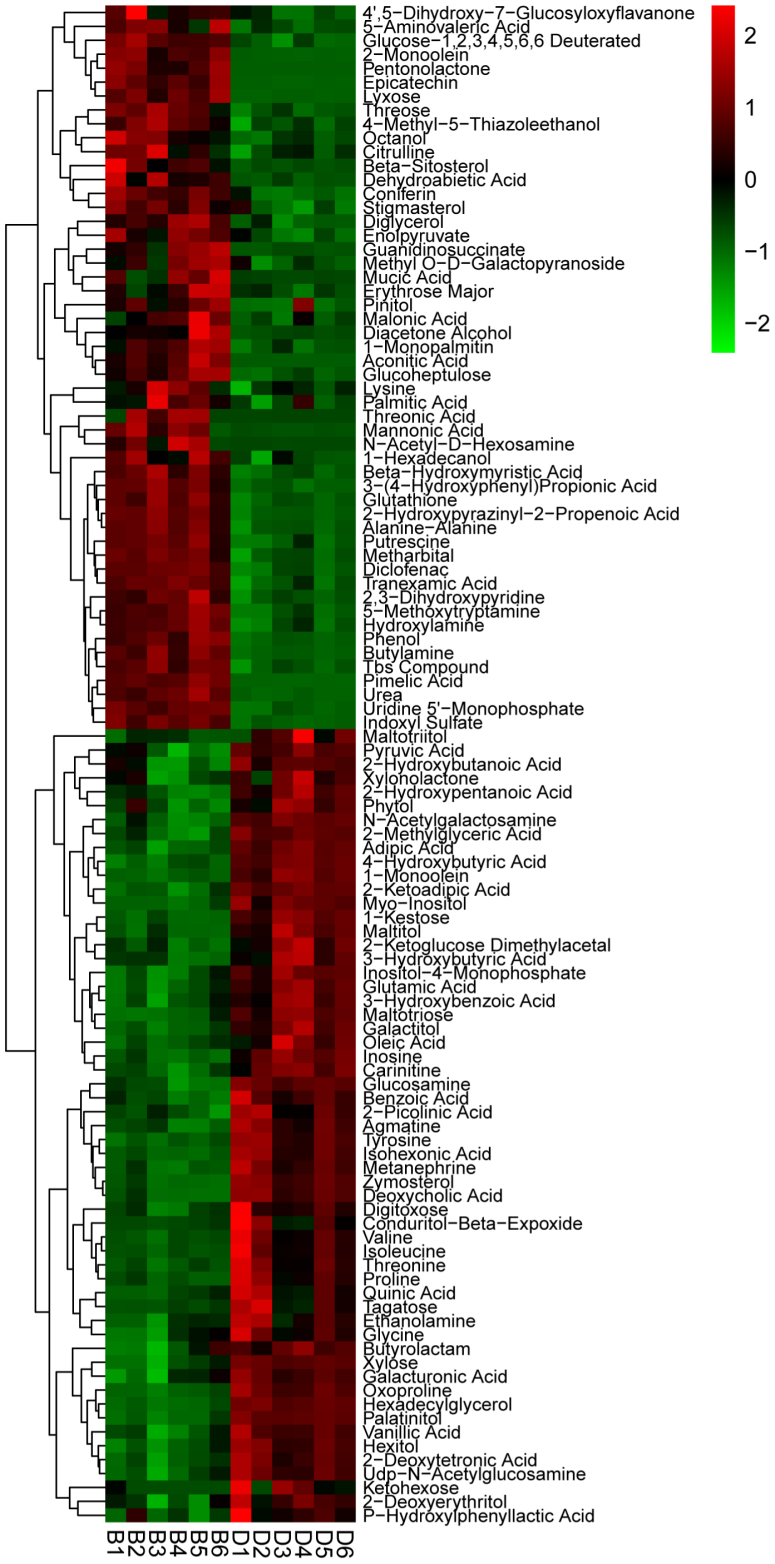
Thermogram analysis of different metabolites in bayberry rhizosphere soil. D and B represent the biochar’s absence and presence in diseased bayberry trees, respectively.

One hundred and nine different metabolites were found in the presence and absence of biochar, mainly including acid, alcohol, ester, amine, amino acid, sterol, sugar, and other secondary metabolites. Of which the contents of 52 metabolites were increased significantly, with a range of 11.48–5538.52%, while the contents of 57 metabolites decreased significantly from 13.32 to 90.37% (Figure 6; Supplementary Table S1). The significantly increased secondary metabolites may be beneficial to plants, interestingly, 11 metabolites had an increased rate of 855.3–5538.52%, specifically aconitic acid, threonic acid, pimelic acid, epicatechin, lyxose, N-acetyl-D-hexosamine, pentonolactone, 2-monoolein, mannonic acid, guanidinosuccinate, mucic acid, etc. On the other hand, the significantly reduced secondary metabolites may be harmful to plants, 36 metabolites had a reduction rate of more than 50%, specifically conduritol β-expoxide, zymosterol, palatinitol, quinic acid, isohexoic acid, deoxycholic acid, maltitol, proline, 1-kestose, and ketohexose, etc.

### 3.8. Analysis of metabolic pathways of differential metabolites

**Figure 7:**
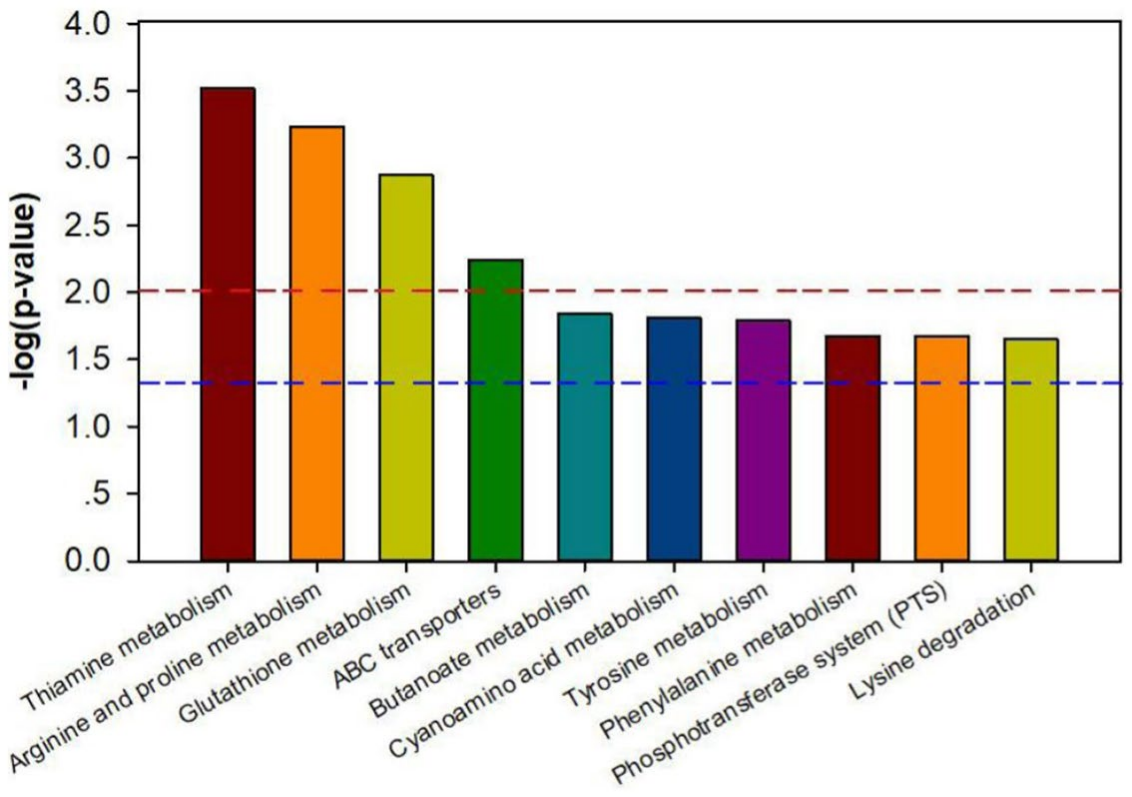
Metabolic pathway enrichment map of different metabolites in bayberry rhizosphere soil of the biochar treatment. The signal pathway is significant when the top of the bar is higher than the blue (*p* < 0.05) or red line (*p* < 0.01).

The results of the Kyoto Encyclopedia of Genes and Genomes (KEGG) database analysis on the pathway enrichment of the differential metabolites of declined diseased trees and biochar treatment showed that the top 10 pathways with the most significant amount of enrichment were thiamine metabolism, arginine, and proline metabolism, glutathione metabolism, ABC transporters, butanoate metabolism, cyanoamino acid metabolism, tyrosine metabolism, phenylalanine metabolism, phosphotransferase system (pts), and lysine degradation (Figure 7).

### 3.9. Correlation of soil bacteria and fungi with metabolites

The bacteria were closely related to the relative contents of the secondary metabolites in the rhizosphere soil at the phyla level (Supplementary Figure S3), at the order level (Supplementary Figure S4). Compared with the correlation between bacterial phyla and secondary metabolites, the correlation between fungal phyla and secondary metabolites in rhizosphere soil is weak, and the positive and negative correlation was not evident with the increase or decrease of the relative content of secondary metabolites (Supplementary Figure S5). Compared with the order of bacteria and secondary metabolites, the order of fungi has a weak correlation with secondary metabolites in rhizosphere soil. Only 4 orders, including Capnodiales, Hymenochaetales, Trechisporales, and Geminibasidiales, had significant correlations with the content of secondary metabolites (Supplementary Figure S6).

**Figure 8:**
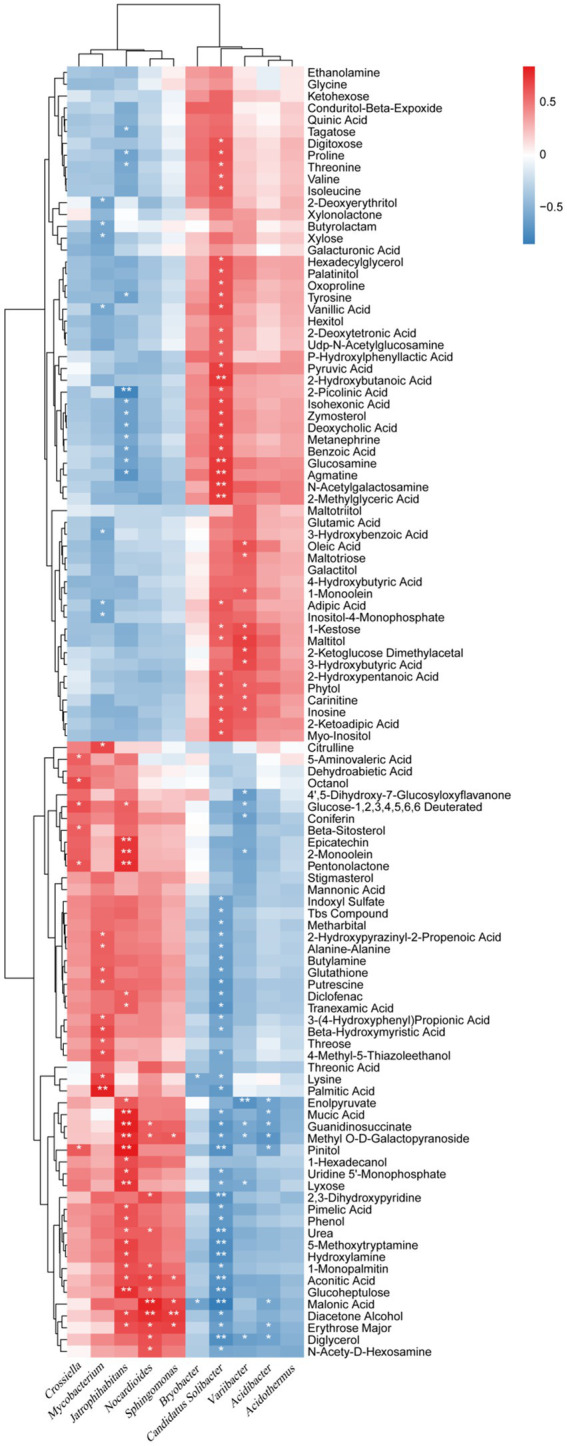
Correlation analysis between bacterial relative abundances at the genus level and the relative metabolite contents of the biochar treatment. ^*^ and ^**^ represent a significant correlation at *p* < 0.05 and *p* < 0.01, respectively. The depth of the orange and blue scale indicated the magnitude of the correlation coefficient. In contrast, the orange darker color has a greater positive correlation, and the blue darker color has a greater negative correlation.

At the genus level, bacteria were closely related to the relative content of secondary metabolites in the rhizosphere soil. Five genera, *Bryobacter*, *Candidatus Solibacter*, *Varribacter*, *Acidibacter*, and *Acidothermus*, were mainly negatively related to 52 metabolites with increased relative content and positively related to 57 secondary metabolites with decreased relative content (Figure 8). Five genera including *Crossiella*, *Mycobacterium*, *Jatrophihabitans*, *Nocardiode*s, and *Sphingomonas* were mainly positively correlated with 52 metabolites with increased relative content and negatively correlated with 57 secondary metabolites with decreased relative content (Figure 8). *Candidatus Solibacter* was the most closely related to the relative content of metabolites, which was significantly negatively related to 35 secondary metabolites with increased relative content, and significantly positively related to 34 secondary metabolites with decreased relative content, accounting for 67.31 and 59.65%, respectively. *Varribacter* was significantly negatively correlated with nine metabolites with increased relative content, and significantly positively correlated with 10 metabolites with decreased relative content, accounting for 17.31 and 17.54%, respectively. *Mycobacterium* was significantly positively correlated with 11 metabolites with increased relative content and negatively correlated with seven metabolites with decreased relative content, accounting for 21.15 and 12.28%, respectively. *Jatrophihabitans* had a significant positive correlation with 24 secondary metabolites with increased relative content and a significant negative correlation with 12 metabolites with decreased relative content, accounting for 46.15 and 21.05%, respectively. *Nocardiodes* were significantly positively correlated with 12 metabolites with increased relative content but not with those with decreased relative content. This data show that bacteria are closely related to the relative content of secondary metabolites at the genus level, among which *Candidatus Solibacter*, *Varribacter*, *Mycobacterium*, and *Jatrophihabitans*, *Nocardiodes* are the most influential.

**Figure 9:**
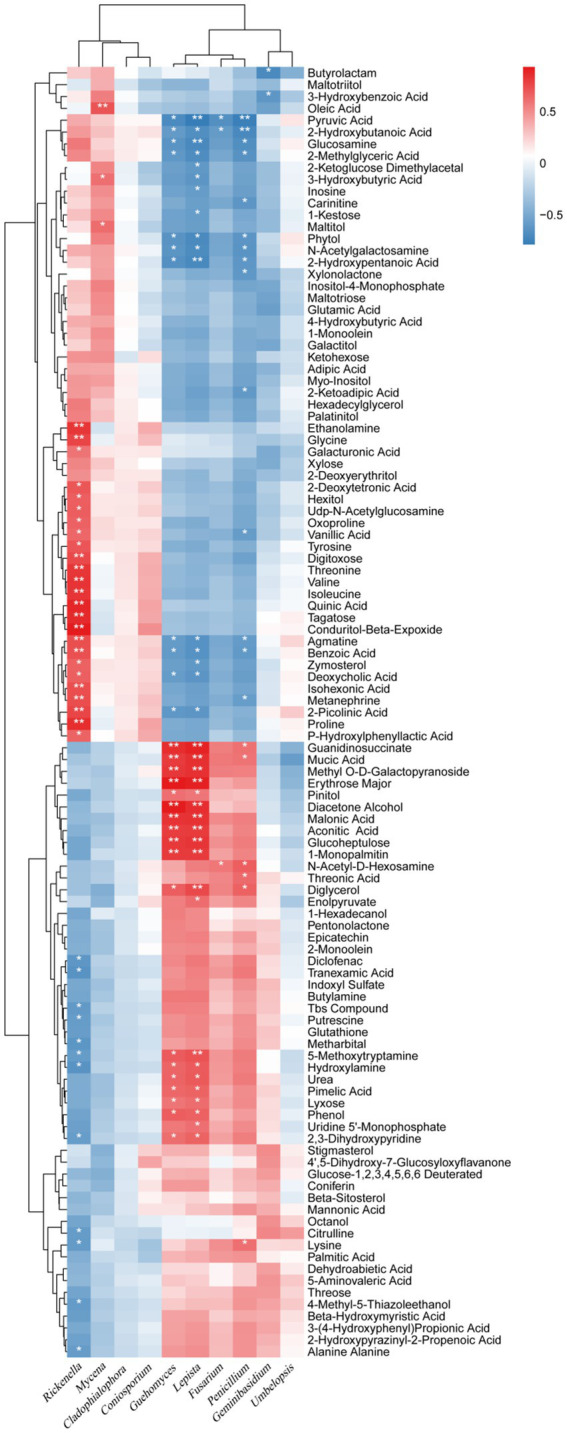
Correlation analysis between the microorganism relative abundances at the fungal genus level and the relative metabolite contents of the biochar treatment. ^*^ and ^**^ represent a significant correlation at *p* < 0.05 and *p* < 0.01, respectively. The depth of the orange and blue scale indicated the magnitude of the correlation coefficient. In contrast, the orange darker color has a greater positive correlation, and the blue darker color has a greater negative correlation.

The correlation between fungi and secondary metabolites in rhizosphere soil was also strong, but slightly lower than that of bacteria. The six fungal genera, including *Geuhomyces*, *Lepista*, *Fusarium*, *Penicillium*, *Geminibasidium*, and *Umbelopsis*, were mainly positively correlated with 52 secondary metabolites with increased relative content and negatively correlated with 57 secondary metabolites with decreased relative content; the four fungal genera including *Rickenella*, *Mycena*, *Cladophialophora*, and *Coniosporium* were mainly negatively correlated with 52 secondary metabolites with increased relative content, and positively correlated with 57 secondary metabolites with decreased relative content (Figure 9). *Lepista* had significant positive correlations with 20 secondary metabolites with increased relative content and a significant negative correlation with 16 secondary metabolites with decreased relative content. *Geuhomyces* had significant positive correlations with 18 secondary metabolites with increased relative content and significant negative correlations with 11 secondary metabolites with decreased relative content. *Penicillium* had significant positive correlations with six secondary metabolites with increased relative content and significant negative correlations with 14 secondary metabolites with decreased relative content. *Rickenella* has significant negative correlations with 12 secondary metabolites with increased relative content and significant positive correlations with 25 secondary metabolites with decreased relative content. This data showed that fungi have strong correlations with the relative content of secondary metabolites at the genus level, among which *Lepista*, *Geuhomyces*, *Penicillium*, and *Rickenella* have the strongest correlation.

## 4. Discussion

This result shows that the application of biochar has a good effect on the rejuvenation of decline diseased trees, which is similar to the treatments of compound fertilizer, fungicide, and humic acid on declined diseased bayberry (Ren et al., 2021b, 2022a,b). In agreement with the result of study, Safaei Khorram et al. (2019) reported that biochar application significantly increased the trunk diameter and new shoots of apple trees. Zhang et al. (2020) found that the application of 3% biochar inhibited the level of chromium toxicity and significantly increased the height, biomass, leaf area, chlorophyll pigment, and photosynthesis of blueberry plants. Fahad et al. (2016) reported that Biochar treatment enhances cucumber growth and yields under high-temperature stress. She et al. (2018) revealed that biochar treatment can reduce the harm of salt stress to crops and improve the indicators such as crop nutrient growth, yield, and quality.

Duan et al. (2019) found that the application of biochar changed the structure of soil microbial bacterial community, which can significantly increase the relative abundance of Acidobacteria, Gemanophyta, Nitrospira, Bacillariaceae, Gemanophytae, and Nitrosaceae. Zhao et al. (2020) have shown that the application of biochar can increase the microbial demand for N, promote the soil N-cycle, including nitrification and denitrification processes, and enhance the microbial fixation of N. In agreement with the result of previous studies, the result of this study also suggest that soil microbial community was significantly affected by biochar, which had a greater effect on the bacterial richness of rhizosphere soil of diseased trees than that of fungi. In contrast, Zhao L. et al. (2019) found that the fungal community is more affected than the bacterial community.

Compared with the diseased trees, the application of biochar caused a 70.36 and 71.35% increase, respectively, in the relative abundance of *Mycobacterium* and *Crossiella* in rhizosphere soil. Interestingly, Pan et al. (2020) reported that *Mycobacterium* strain Mya-zh01facilitats plant growth and seed germination in *Doritaenopsis* by IAA biosynthesis, while Gonzalez-Pimentel et al. (2022) revelaed the bacteriostatic function of *Crossiella*. Thus, *Mycobacterium* and *Crossiella* may be beneficial to the growth of diseased bayberry. On the other hand, the relative abundance of *Acidothermus*, *Bryobacter*, and *Acidibacter* was decreased by 16.38, 13.06, and 26.41%, respectively, indicating they may be harmful to the growth of diseased bayberry. Indeed, the functions of *Acidothermus*, *Bryobacter*, and *Acidibacter* on plants are rarely reported.

Compared with the diseased trees, the application of biochar caused a 66.09 and 98.14% increase, respectively, in the relative abundance of *Geminibasidium* and *Fusarium*. Interestingly, *Geminibasidium* species have been reported to be heat tolerant and xerotolerant, which makes this genus richness in soil (Borrell et al., 2017). *Fusarium* is a kind of soil-borne fungus, often used as the plant pathogen, which can cause root rot and *Fusarium* disease in wheat, barley, and other small grain crops, pollute grains with mycotoxins and cause significant losses in yield and quality (Coleman, 2016; LeBlanc et al., 2017; Song et al., 2018; Xi et al., 2019). The significant increase in the number of *Fusarium* species by biochar in this study, revealing the complexity of its function. On the other hand, the relative abundance of *Cladophialophora*, *Mycena*, and *Rickenella*’s were significantly reduced by 16.74, 94.70, and 99.79%, respectively, indicating they maybe be harmful to the bayberry. Indeed, the functions of *Cladophialophora*, *Mycena*, and *Rickenella* on plants have been rarely reported.

Previous studies have shown that biochar amendments can increase both plant nutrient uptake and crop production in nutrient-deficient soil (Solaiman et al., 2019), while the original biochars could decrease available soil P through P adsorption (Li et al., 2020). Applying biochar can increase eucalyptus root soil’s organic carbon and N, P, and K content (Daun et al., 2020). However, this result is different from the previous research results that applying biochar + compost treatment can increase apple tree rhizosphere soil’s total organic carbon and available P content (Safaei Khorram et al., 2019), which may be because bayberry’s utilization mechanism differs from apple and eucalyptus’s available phosphorus and potassium. This data indicated that biochar application had a better effect on improving trace elements in the rhizosphere soil of declined bayberry.

In contrast with the result of this study, the alkali hydrolyzable nitrogen, phosphorus, potassium, and exchangeable calcium contribution rate to bacteria after humic acid treatment was greater than that of fungi (Ren et al., 2022a). The pH, organic matter, alkali hydrolyzable nitrogen, available phosphorus, and exchangeable magnesium after fungicide treatment contribute more to bacteria than fungi (Ren et al., 2022b). The contribution rate of organic matter, available phosphorus, and exchangeable calcium to bacteria after fertilizer treatment were higher than those of fungi (Ren et al., 2021b). These conflict results may be mainly due to the difference in soil treatment measures, material characteristics and improvement mechanisms, suggesting that a balance of nutrients may be best for microbial growth. Overall, the result of this study reveals the complexity of the relationship between microbial growth and soil nutrient elements.

Generally, few reports were available about the functions of the 11 significantly increased substances in plants and microorganisms. However, in agreement with the result of this study, the eight substances, including aconitic acid, threonic acid, epicatechin, lyxose, N-acetyl-D-hexosamine, pentonolactone, 2-monoolein, and guanidinosuccinate have also been increased significantly in the decline bayberry rhizosphere soil treated with humic acid and bio-organic fertilizer (Ren et al., 2022a,b), which indicates that there are some similar mechanisms in different soil improvement measures. Interestingly, aconitic acid has been found to play various biological roles within cells as an intermediate in the tricarboxylic acid cycle. It confers unique survival advantages to some plants as an antifeedant, antifungal, and means of storing fixed pools of carbon. Furthermore, aconitic acid has also been reported as an inhibitor of fermentation, an anti-inflammatory, and a possible nematicide (Bruni and Klasson, 2022).

Similarly, few reports were available about the functions of the 36 significantly reduced substances in plants and microorganisms. However, five substances, including zymosterol, palatinitol, deoxycholic acid, proline, and 1-kestose, have also been significantly decreased in the decline bayberry rhizosphere soil treated with humic acid and bio-organic fertilizer (Ren et al., 2022a,b), which indicates that there are some similar mechanisms in different soil improvement measures. Interestingly, proline has been found to be a multifunctional amino acid involved in plant adaptation to environmental constraints (Ben et al., 2012). Organic acid is a kind of important metabolite secreted into rhizosphere soil by plant roots, which can improve the availability of soil nutrients and affected by soil environmental factors (Clarholm et al., 2015). Carbohydrates are energy sources of microorganisms, which can provide metabolites for various anabolic pathways, or are general chemotactic substances (Zhu et al., 2016; Salcedo-Vite et al., 2019). Sugar metabolism of plants has the functions of ensuring normal plant growth and development by providing energy, building carbon skeleton, and regulating osmotic pressure, gene regulation, and signal transmission (Smeekens, 2000). Amino acids are substrates for protein synthesis, which can induce plant growth, and promote plant recovery from stress to normal metabolism and osmotic balance by regulating ion transport and detoxify heavy metals. Leucine can promote plant growth and has a unique role in photosynthesis and regulation, while tyrosine can regulate plant root tips and maintain root cells (Huang et al., 2014; Zhao F. et al., 2019).

In this study, biochar caused a significant increase in the relative contents of threonic acid, pimelic acid, mannonic acid, mucic acid, and 3-(4-hydroxyphenyl) propionic acid in rhizosphere soil of declined bayberry trees. This increase may be because biochar can promote the metabolism level of declined bayberry by improving the availability of root-soil nutrients. The relative contents of deoxycholic acid, 2-hydroxybutanoic acid, and adipic acid were reduced significantly. This may be because biochar can improve soil environment, and help the growth of declined bayberry by promoting the decomposition of the above substances in the tree body. The content of carbohydrate compounds, such as ketohexose, maltitol, and maltotriose were reduced significantly. This may be because that biochar can promote the growth of beneficial microorganisms, resulting in the increase in the demand for carbon sources, and the improvement in the utilization rate of carbohydrates in the soil. It may also be because that biochar can promote the growth and development of the diseased bayberry by stimulating the change of sugar metabolism in bayberry tree. The relative contents of isoleucine and tyrosine were reduced significantly, possibly due to the increased utilization rate of isoleucine and tyrosine in declined bayberry.

Compared with the 10 significantly different pathways caused by humic acid treatment on the soil of diseased trees, there were the common six pathways; namely, arginine and proline metabolism, glutathione metabolism, ABC transporters, butanoate metabolism, cyanoamino acid metabolism, and phosphotransferase system (Ren et al., 2022a). Compared with the seven significant difference pathways caused by the compound fertilizer treatment on the soil of diseased trees, cyanoamino acid metabolism, and phenylalanine metabolism were common. No significant difference was observed in pathway caused by biological organic fertilizer on the soil of diseased trees (Ren et al., 2021b). Compared with the four significantly different pathways caused by the fungicide treatment on the soil of diseased trees, arginine and proline metabolism, and butanoate metabolism were common (Ren et al., 2022a).

Thiamine is the precursor of thiamine diphosphate, which is a coenzyme of more than 20 kinds of characteristic enzymes. These enzymes not only participate in the cell bioenergy process leading to ATP synthesis but also involved in the biosynthesis of pentose (required for nucleotide synthesis), amino acid, and other organic compounds of cell metabolism (Tylicki and Siemieniuk, 2011). Arginine and proline are essential components of plant amino acids, while arginine and proline metabolism play a good role in cold, drought, and salt tolerance. Arginine has the functions of ammonia detoxification, hormone secretion, immune regulation, and nitrogen storage, while proline is the energy to restore growth and plays a role in maintaining the balance of protoplasm and environmental penetration by preventing water loss (Appleton, 2002). Glutathione is a simple antioxidant which can reduce the oxidative damage caused by drought by regulating the gene expression of key enzymes, thus playing a protective role (Lou et al., 2018). ABC transporters found in all living organisms constitute one of the largest and most ancient protein superfamilies, which function as molecular machines by coupling ATP binding, hydrolysis, and phosphate release to translocate diverse substrates across membranes (Thomas and Tampe, 2020). Therefore, biochar may promote the root growth of diseased bayberry trees through arginine and proline metabolism and improve the resistance of bayberry to cold, drought, salt and oxidative stresses.

## 5. Conclusion

Result from this study clearly indicated that biochar could improve the vigor and fruit quality of diseased trees, and the diversity and species number of microbial communities in the rhizosphere soil. Compared with the diseased trees, the relative abundance of *Acidothermus*, *Bryobacter*, *Acidibacter*, *Cladophialophora*, *Mycena*, and *Rickenella* were significantly decreased, while the relative abundance of *Mycobacterium*, *Crossiella*, *Geminibasidium*, and *Fusarium* were significantly increased by biochar in rhizosphere soil. The RDA of microbial communities and soil characteristics revealed that the pH, organic matter, alkali hydrolyzable nitrogen, available phosphorus, available potassium, exchangeable calcium, and exchangeable magnesium significantly affected the composition of bacterial and fungal communities in bayberry rhizosphere soil. Biochar greatly influenced the metabolomics distribution of diseased bayberry rhizosphere soils. Among the 109 common metabolites, the contents of 52 metabolites such as aconitic acid, threonic acid, pimelic acid, epicatechin, and lyxose were increased significantly, while the contents of 57 metabolites such as conduritol β-expoxide, zymosterol, palatinitol, quinic acid, and isohexoic acid were decreased significantly. Biochar caused change in 10 metabolic pathways, including thiamine metabolism, arginine and proline metabolism, glutathione metabolism, ABC transporters, butanoate metabolism, cyanoamino acid metabolism, tyrosine metabolism, phenylalanine metabolism, phosphotransferase system, and lysine degradation. There was a significant correlation between the relative content of microbial species and the content of secondary metabolites in rhizosphere soil at the levels of phylum, order, and genus. Overall, this study highlighted the great influence of biochar in decline disease by regulating microbial community, physical and chemical properties, and secondary metabolites in the bayberry rhizosphere soil, which provided a novel strategy for managing bayberry decline disease.

## Data availability statement

The datasets presented in this study can be found in online repositories. The names of the repository/repositories and accession number(s) can be found at: https://www.ncbi.nlm.nih.gov/, CRA001372 and https://www.ncbi.nlm.nih.gov/, SUB12144729.

## Author contributions

HR, HW, XQ, QW, and BL: conceptualization and methodology. HR, HG, HZ, and KR: data curation. HR, HW, KR, and JG: investigation. HR, MS, GL, and BL: writing—original draft. HZ, HR, MS, GL, QW, and BL: writing—review and editing. All authors contributed to the article and approved the submitted version.

## Funding

This work was supported by the Key R&D projects in Zhejiang Province (2021C02009, 2019C02038, and 2020C02001) and Zhejiang Major Agricultural Technology Collaborative Promotion Plan Project (2022XTTGGP04).

## Conflict of interest

The authors declare that the research was conducted in the absence of any commercial or financial relationships that could be construed as a potential conflict of interest.

## Publisher’s note

All claims expressed in this article are solely those of the authors and do not necessarily represent those of their affiliated organizations, or those of the publisher, the editors and the reviewers. Any product that may be evaluated in this article, or claim that may be made by its manufacturer, is not guaranteed or endorsed by the publisher.
