# Supplementary material for: Improvement effect of biochar on soil microbial community structure and metabolites of decline disease bayberry
Source: Front Microbiol. 2023 May 12;14:1154886. doi: 10.3389/fmicb.2023.1154886 (PMC10275294; doi:10.3389/fmicb.2023.1154886)
Supplement: Supplementary file 1 [file Data_Sheet_1.docx]

**Supplementary data**

**Table S1.** The relative contents of the metabolites in rhizosphere soil were changed by biochar on the decline disease bayberry trees.

| Metabolite name |  | Relative content |  |  | Relative content |
| --- | --- | --- | --- | --- | --- |
| Aconitic Acid | B | 43.54*±3.51 | Threonic Acid | B | 9.32*±1.76 |
|  | D | 0.77±0.01 |  | D | 0.29±0.02 |
| Pimelic Acid | B | 11.67*±1.27 | Epicatechin | B | 10.71*±1.06 |
|  | D | 0.37±0.03 |  | D | 0.35±0.05 |
| Lyxose | B | 10.38*±2.07 | N-Acetyl-D-Hexosamine | B | 6.63*±0.43 |
|  | D | 0.42±0.09 |  | D | 0.29±0.05 |
| Pentonolactone | B | 41.56*±4.67 | 2-Monoolein | B | 7.27*±1.55 |
|  | D | 1.91±0.03 |  | D | 0.48±0.06 |
| Mannonic Acid | B | 9.23*±1.55 | Guanidinosuccinate | B | 3.85*±0.80 |
|  | D | 0.69±0.15 |  | D | 0.30±0.03 |
| Mucic Acid | B | 2.63*±1.76 | Pinitol | B | 15.74*±1.32 |
|  | D | 0.28±0.07 |  | D | 5.54±0.69 |
| 4',5-Dihydroxy-7-Glucosyloxyflavanone | B | 8.32*±3.12 | Dehydroabietic Acid | B | 5.22*±0.56 |
|  | D | 2.99±1.35 |  | D | 2.00±0.21 |
| 5-Methoxytryptamine | B | 131.82*±12.92 | Diclofenac | B | 8.57*±0.08 |
|  | D | 58.12±5.05 |  | D | 4.61±0.44 |
| Coniferin | B | 0.73*±0.06 | Diacetone Alcohol | B | 10.12*±2.79 |
|  | D | 0.41±0.06 |  | D | 5.62±0.22 |
| Glucoheptulose | B | 8.70*±0.25 | Uridine 5'-Monophosphate | B | 0.61*±0.02 |
|  | D | 4.88±0.37 |  | D | 0.35±0.02 |
| Beta-Sitosterol | B | 4.07*±0.92 | Octanol | B | 1.37*±0.26 |
|  | D | 2.33±0.08 |  | D | 0.86±0.09 |
| Hydroxylamine | B | 35.44*±2.11 | Indoxyl Sulfate | B | 1.14*±0.05 |
|  | D | 22.48±2.52 |  | D | 0.73±0.02 |
| Glucose-1,2,3,4,5,6,6 Deuterated | B | 0.56*±0.03 | Butylamine | B | 1.54*±0.09 |
|  | D | 0.37±0.03 |  | D | 1.05±0.02 |
| Metharbital | B | 8.76*±0.43 | 1-Hexadecanol | B | 1.80*±0.15 |
|  | D | 6.03±0.47 |  | D | 1.25±0.16 |
| Phenol | B | 33.26*±2.04 | Stigmasterol | B | 0.49*±0.04 |
|  | D | 23.03±0.64 |  | D | 0.35±0.05 |
| Threose | B | 0.44*±0.04 | Urea | B | 237.75*±11.08 |
|  | D | 0.31±0.02 |  | D | 170.82±1.40 |
| Diglycerol | B | 0.49*±0.04 | 3-(4-Hydroxyphenyl) Propionic Acid | B | 38.7*±1.95 |
|  | D | 0.36±0.02 |  | D | 27.99±0.62 |
| Glutathione | B | 1.60*±0.02 | Alanine-Alanine | B | 181.4*±7.94 |
|  | D | 1.16±0.02 |  | D | 132.12±6.12 |
| Enolpyruvate | B | 3.15*±0.32 | Putrescine | B | 301.32*±13.29 |
|  | D | 2.32±0.21 |  | D | 223.48±12.81 |
| 2,3-Dihydroxypyridine | B | 0.66*±0.04 | 2-Hydroxypyrazinyl-2-Propenoic Acid | B | 168.06*±6.69 |
|  | D | 0.49±0.02 |  | D | 124.87±3.48 |
| Tbs Compound | B | 5.26*±0.21 | 4-Methyl-5-Thiazoleethanol | B | 0.45*±0.03 |
|  | D | 3.92±0.18 |  | D | 0.34±0.02 |
| 1-Monopalmitin | B | 51.84*±4.33 | Methyl O-D-Galactopyranoside | B | 13.18*±1.70 |
|  | D | 40.07±1.86 |  | D | 10.22±1.01 |
| Palmitic Acid | B | 10.38*±1.41 | Tranexamic Acid | B | 1.40*±0.03 |
|  | D | 8.25±1.01 |  | D | 1.14±0.05 |
| Lysine | B | 0.70*±0.09 | Beta-Hydroxymyristic Acid | B | 4.12*±0.17 |
|  | D | 0.57±0.05 |  | D | 3.42±0.08 |
| Citrulline | B | 7.05±0.73 | Malonic Acid | B | 0.20±0.02 |
|  | D | 5.88±0.39 |  | D | 0.17±0.01 |
| Erythrose Major | B | 1.77*±0.17 | 5-Aminovaleric Acid | B | 0.45*±0.02 |
|  | D | 1.54±0.05 |  | D | 0.41±0.01 |
| Xylonolactone | B | 0.96^#^±0.07 | 2-Hydroxybutanoic Acid | B | 0.87^#^±0.09 |
|  | D | 1.11±0.09 |  | D | 1.08±0.04 |
| 2-Deoxyerythritol | B | 2.38^#^±0.30 | P-Hydroxylphenyllactic Acid | B | 0.34^#^±0.05 |
|  | D | 3.10±0.30 |  | D | 0.45±0.07 |
| Butyrolactam | B | 2.26^#^±0.22 | Glycine | B | 5.73^#^±0.98 |
|  | D | 3.09±0.20 |  | D | 8.22±1.24 |
| Phytol | B | 0.60^#^±0.04 | 2-Picolinic Acid | B | 1.60^#^±0.19 |
|  | D | 0.92±0.13 |  | D | 2.46±0.35 |
| Vanillic Acid | B | 1.15^#^±0.17 | Xylose | B | 0.65^#^±0.10 |
|  | D | 1.81±0.15 |  | D | 1.04±0.03 |
| Digitoxose | B | 1.82^#^±0.24 | Ethanolamine | B | 3.11^#^±0.32 |
|  | D | 2.96±0.27 |  | D | 5.11±0.55 |
| Inosine | B | 0.46^#^±0.04 | Galacturonic Acid | B | 0.66^#^±0.02 |
|  | D | 0.79±0.07 |  | D | 1.13±0.09 |
| 2-Ketoadipic Acid | B | 1.96^#^±0.24 | Benzoic Acid | B | 3.46^#^±0.51 |
|  | D | 3.53±0.12 |  | D | 6.30±0.94 |
| Adipic Acid | B | 0.56^#^±0.08 | 2-Methylglyceric Acid | B | 0.53^#^±0.10 |
|  | D | 1.04±0.05 |  | D | 1.00±0.05 |
| Myo-Inositol | B | 26.28^#^±2.31 | Udp-N-Acetylglucosamine | B | 0.89^#^±0.21 |
|  | D | 51.82±5.53 |  | D | 1.76±0.11 |
| Oleic Acid | B | 0.39^#^±0.06 | Pyruvic Acid | B | 1.80^#^±0.16 |
|  | D | 0.77±0.18 |  | D | 3.67±0.30 |
| Agmatine | B | 0.61^#^±0.10 | Threonine | B | 0.58^#^±0.07 |
|  | D | 1.26±0.20 |  | D | 1.23±0.29 |
| 3-Hydroxybenzoic Acid | B | 3.86^#^±0.14 | 2-Deoxytetronic Acid | B | 0.95^#^±0.09 |
|  | D | 8.23±0.48 |  | D | 2.02±0.28 |
| Inositol-4-Monophosphate | B | 1.00^#^±0.26 | Glutamic Acid | B | 0.82^#^±0.19 |
|  | D | 2.13±0.24 |  | D | 1.76±0.14 |
| N-Acetylgalactosamine | B | 1.95^#^±0.17 | 4-Hydroxybutyric Acid | B | 3.69^#^±0.48 |
|  | D | 4.24±0.23 |  | D | 8.08±0.47 |
| Glucosamine | B | 0.47^#^±0.10 | 2-Hydroxypentanoic Acid | B | 0.70^#^±0.02 |
|  | D | 1.02±0.06 |  | D | 1.65±0.27 |
| Metanephrine | B | 0.18^#^±0.03 | 2-Ketoglucose Dimethylacetal | B | 1.77^#^±0.14 |
|  | D | 0.42±0.07 |  | D | 4.44±0.15 |
| Hexadecylglycerol | B | 1.03^#^±0.12 | Maltotriose | B | 0.82^#^±0.03 |
|  | D | 2.68±0.13 |  | D | 2.20±0.31 |
| 3-Hydroxybutyric Acid | B | 10.96^#^±1.66 | Oxoproline | B | 14.32^#^±1.34 |
|  | D | 30.44±3.12 |  | D | 41.30±4.88 |
| Valine | B | 1.50^#^±0.25 | Maltotriitol | B | 4.23^#^±0.73 |
|  | D | 4.38±0.35 |  | D | 12.58±1.33 |
| Galactitol | B | 6.97^#^±0.70 | Hexitol | B | 1.48^#^±0.67 |
|  | D | 21.08±3.78 |  | D | 4.61±0.69 |
| Carinitine | B | 1.71^#^±0.12 | Isoleucine | B | 0.64^#^±0.09 |
|  | D | 5.33±0.44 |  | D | 2.21±0.26 |
| Tagatose | B | 13.33^#^±2.66 | 1-Monoolein | B | 0.51^#^±0.07 |
|  | D | 47.61±2.07 |  | D | 1.91±0.25 |
| Tyrosine | B | 0.43^#^±0.10 | Conduritol-Beta-Expoxide | B | 0.50^#^±0.09 |
|  | D | 1.69±0.12 |  | D | 2.05±0.15 |
| Zymosterol | B | 2.06^#^±0.25 | Palatinitol | B | 2.90^#^±0.10 |
|  | D | 8.71±1.22 |  | D | 12.43±0.74 |
| Quinic Acid | B | 0.50^#^±0.06 | Isohexonic Acid | B | 1.22^#^±0.31 |
|  | D | 2.15±0.22 |  | D | 5.88±0.58 |
| Deoxycholic Acid | B | 2.67^#^±0.44 | Maltitol | B | 1.41^#^±0.17 |
|  | D | 14.16±2.26 |  | D | 8.20±0.84 |
| Proline | B | 1.01^#^±0.46 | 1-Kestose | B | 97.58^#^±8.29 |
|  | D | 5.97±0.37 |  | D | 853.40±43.36 |
| Ketohexose | B | 1.13^#^±0.20 |  |  |  |
|  | D | 11.78±1.88 |  |  |  |


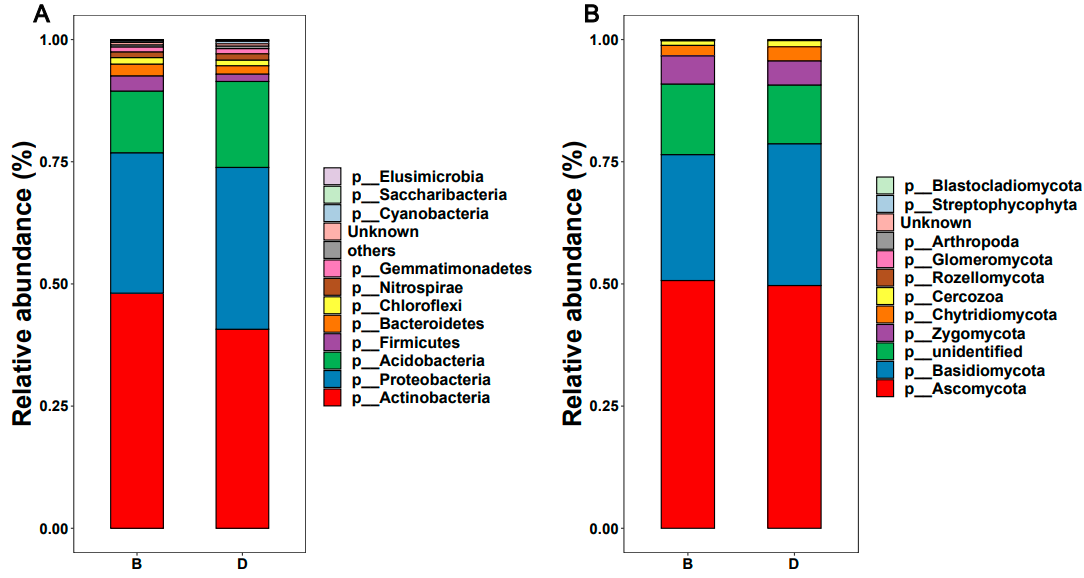


**Figure S1**. Relative abundance of bacteria (A) and fungi (B) at the phylum level. D and B represent the absence and presence of biochar in diseased bayberry trees, respectively.


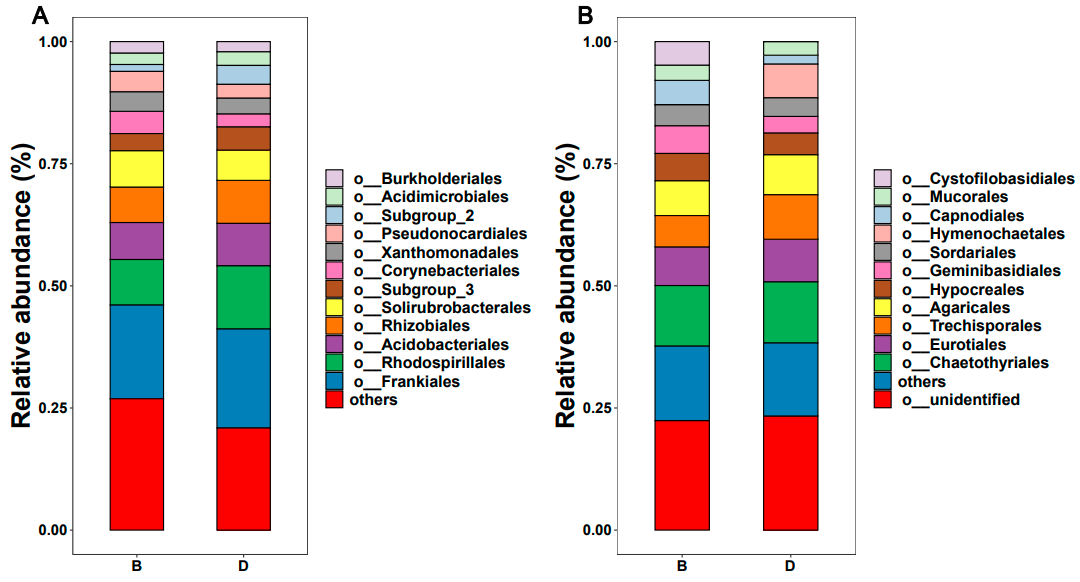


**Figure S2.** Relative abundance of bacteria (A) and fungi (B) at the order level. D and B represent the absence and presence of biochar in diseased bayberry trees, respectively.


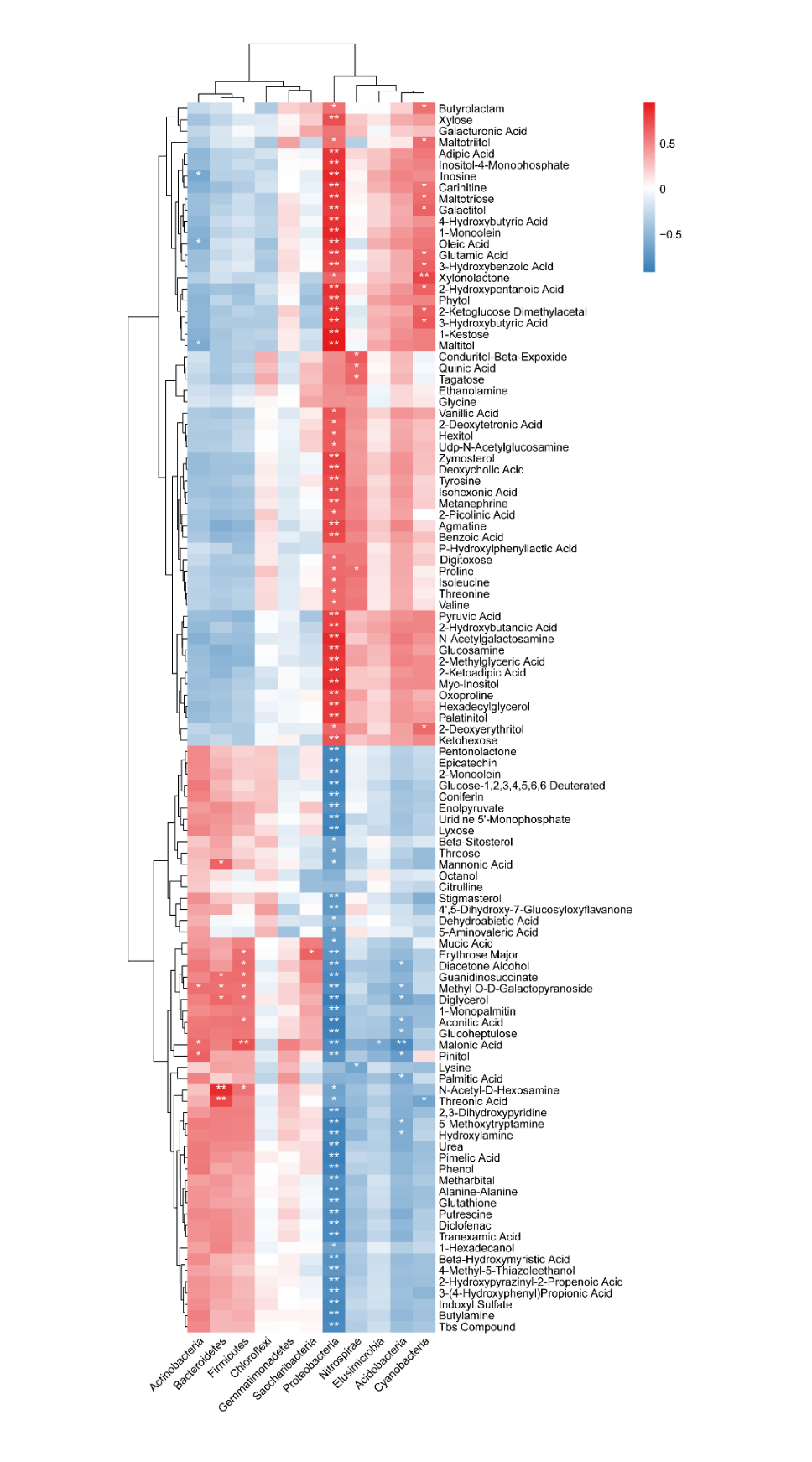


**Figure S3.** Correlation analysis between the microorganism relative abundances at the bacterial phylum level and the relative metabolite contents of the biochar treatment. * and ** represents a significant correlation at *p* < 0.05 and *p* < 0.01, respectively. The depth of the orange and blue scale indicated the magnitude of the correlation coefficient. In contrast, the orange darker color has a greater positive correlation, and the blue darker color has a greater negative correlation.


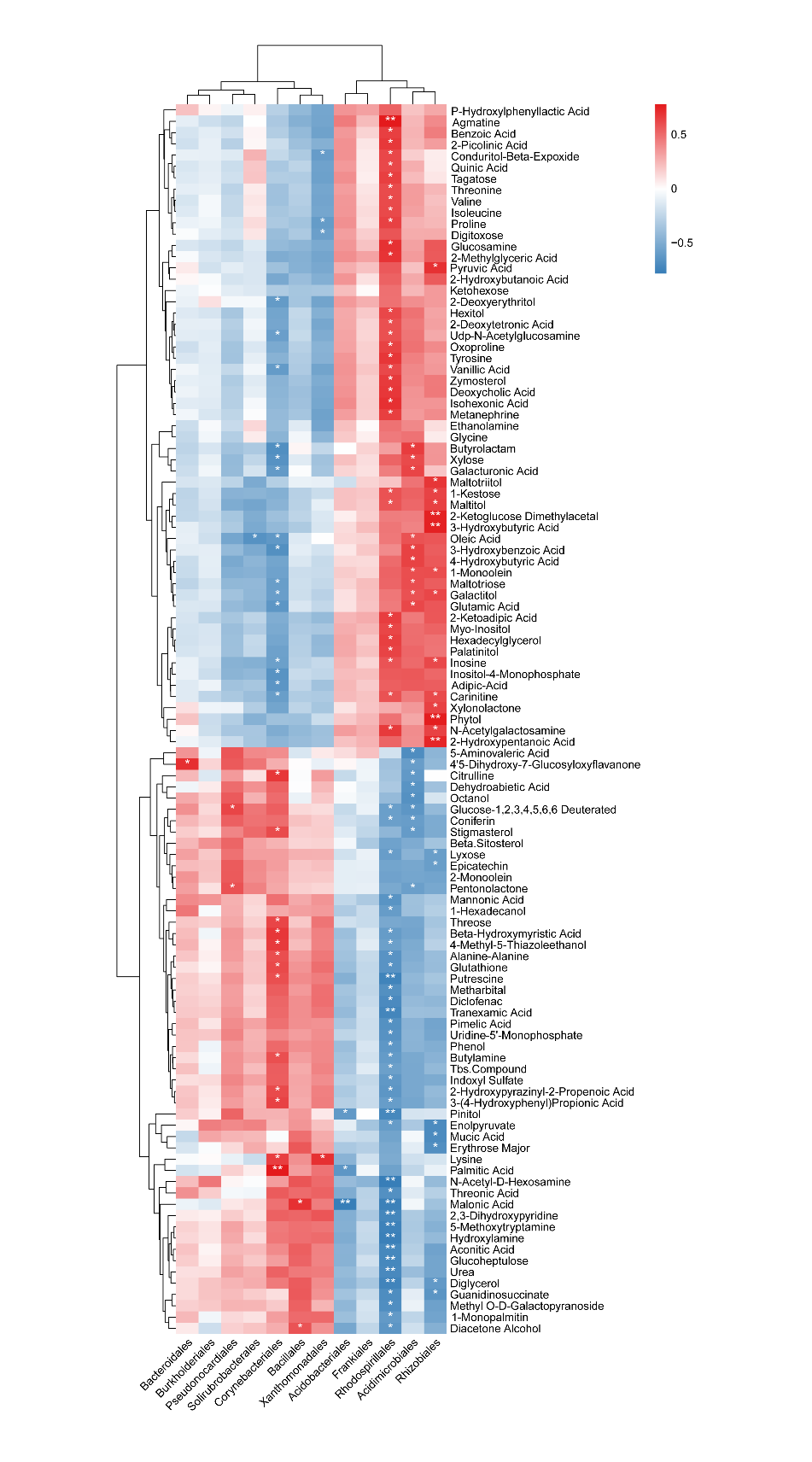


**Figure S4.** Correlation analysis between the microorganism relative abundances at the bacterial order level and the relative metabolite contents of the biochar treatment. * and ** represents a significant correlation at *p* < 0.05 and *p* < 0.01, respectively. The depth of the orange and blue scale indicated the magnitude of the correlation coefficient. In contrast, the orange darker color has a greater positive correlation, and the blue darker color has a greater negative correlation.


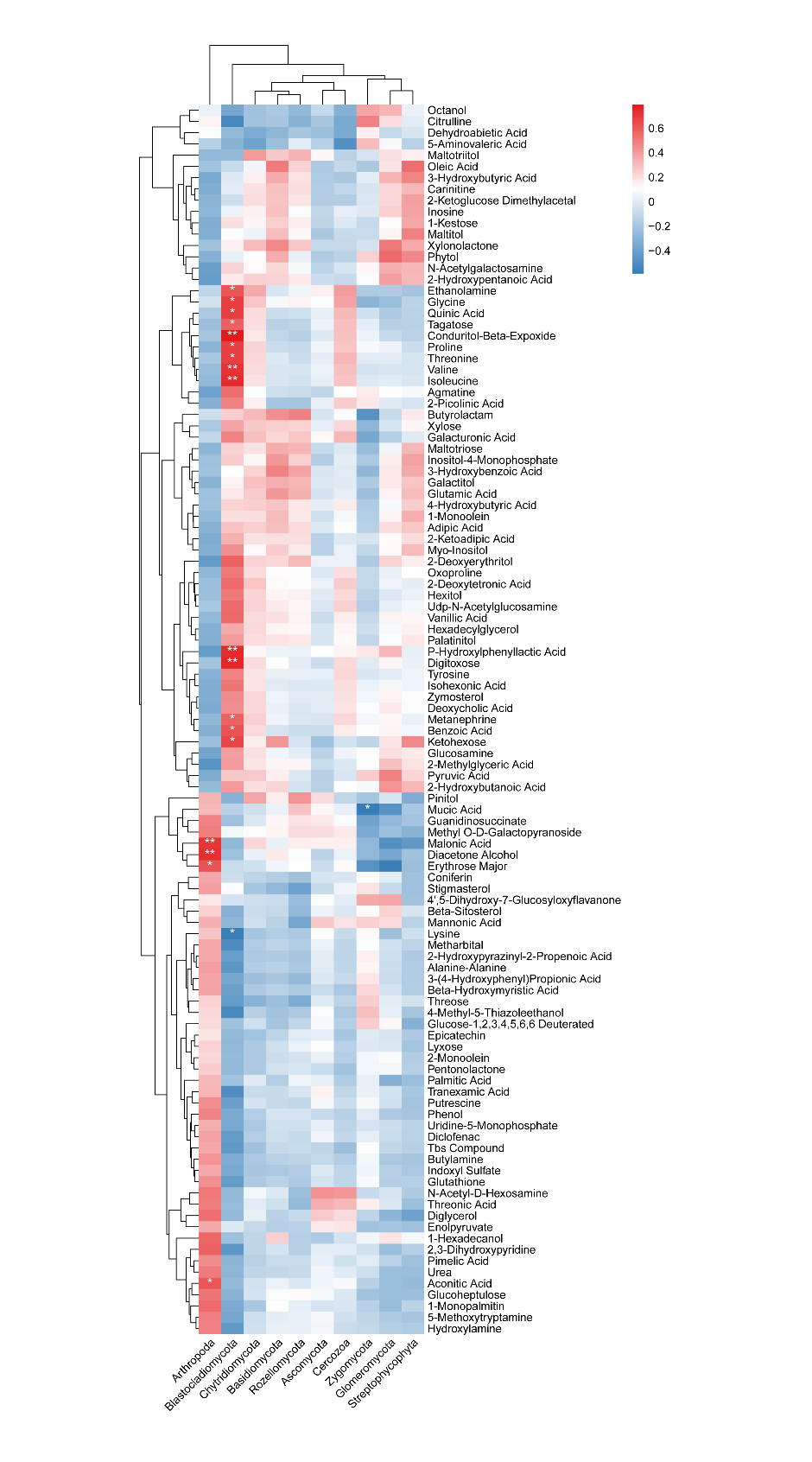


**Figure S5.** Correlation analysis between the microorganism relative abundances at the fungal phylum level and the relative metabolite contents of the biochar treatment. * and ** represents a significant correlation at *p* < 0.05 and *p* < 0.01, respectively. The depth of the orange and blue scale indicated the magnitude of the correlation coefficient. In contrast, the orange darker color has a greater positive correlation, and the blue darker color has a greater negative correlation.


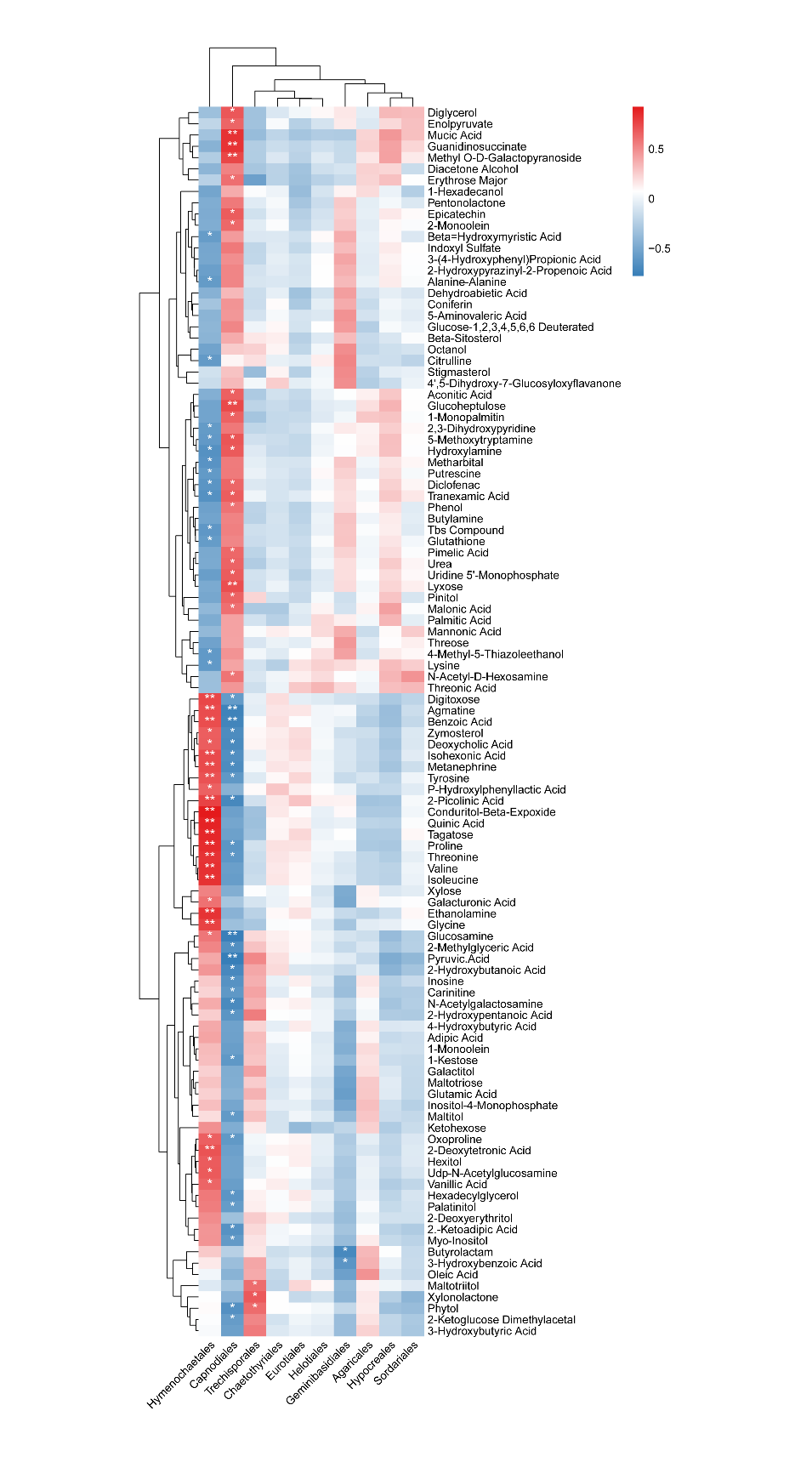


**Figure S6.** Correlation analysis between the microorganism relative abundances at the fungal order level and the relative metabolite contents of the biochar treatment. * and ** represents a significant correlation at *p* < 0.05 and *p* < 0.01, respectively. The depth of the orange and blue scale indicated the magnitude of the correlation coefficient. In contrast, the orange darker color has a greater positive correlation, and the blue darker color has a greater negative correlation.
